# Predictive modeling for the adsorptive and photocatalytic removal of phenolic contaminants from water using artificial neural networks

**DOI:** 10.1016/j.heliyon.2024.e37951

**Published:** 2024-09-20

**Authors:** Shahzar Hafeez, Ayesha Ishaq, Azeem Intisar, Tariq Mahmood, Muhammad Imran Din, Ejaz Ahmed, Muhammad Rizwan Tariq, Muhammad Amin Abid

**Affiliations:** a Centre for Inorganic Chemistry, School of Chemistry, University of the Punjab, 54590, Pakistan; b Centre for Physical Chemistry, School of Chemistry, University of the Punjab, 54590, Pakistan; c Centre for High Energy Physics, University of the Punjab, 54590, Pakistan; d Centre for Organic Chemistry, School of Chemistry, University of the Punjab, 54590, Pakistan; e Department of Food Sciences, University of the Punjab, 54590, Pakistan; f Department of Chemistry, University of Sahiwal, Sahiwal, Pakistan

**Keywords:** Adsorption, Artificial intelligence, Artificial neural network models, Phenolic contaminants, Photocatalysis, Wastewater treatment

## Abstract

Numerous harmful phenolic contaminants are discharged into water that pose a serious threat to environment where two of the most important purification methodologies for the mitigation of phenolic contaminants are adsorption and photocatalysis. Besides cost, each process has drawbacks in terms of productivity, environmental impact, sludge creation, and the development of harmful by-products. To overcome these limitations, the modeling and optimization of water treatment methods is required. Artificial Intelligence (AI) is employed for the interpretation of treatment-based processes due to powerful learning, simplicity, high estimation accuracy, effectiveness, and improvement of process efficiency where artificial neural networks (ANNs) are most frequently employed for predicting and analyzing the efficiency of processes applied for the mitigation of these phenolic contaminants from water. ANNs are superior to conventional linear regression models because the latter are incapable of dealing with non-linear systems. ANNs can also reduce the operational cost of treating phenol-contaminated water. A correlation coefficient of >0.99 can be achieved using ANN with enhanced phenol mitigation percentage accuracy generally ranging from 80 % to 99.99 %. Using ANN optimization, the maximum phenol mitigation efficiencies achieved were 99.99 % for phenol, 99.93 % for bisphenol A, 99.6 % for nonylphenol, 97.1 % for 2-nitrophenol, 96.6 % for 4-chlorophenol and 90 % for 2,6-dichlorophenol. In numerous ANN models, Levenberg-Marquardt backpropagation algorithm for training was employed using MATLAB software. This study overviews their employment and application for optimization and modeling of removal processes and explicitly discusses the important input and output parameters necessary for better performance of the system. The comparison of ANNs with other AI techniques revealed that ANNs have better predictability for mitigation of most of the phenolic contaminants. Furthermore, several challenges and future prospects have also been discussed.

## Highlights

• Numerous sources and harmful effects of various phenolic pollutants.
• Inclusion of robust materials for effective removal of contaminants via adsorption and photocatalytic methodologies.
• Critical discussion of ANNs and comparison with other machine learning techniques.
• Elaboration of various challenges and future prospects.

## 1 Introduction

The significant increase in global population and industrialization throughout the 20th century has led to widespread environmental degradation. Each day, approximately 2 million tons of industrial and agricultural waste are dumped into waterways [1]. Various types of emerging pollutants are present in wastewater [2] and the release of these harmful substances into the ecosystem is well-known for causing water pollution, leading to more stringent regulations on wastewater discharges. The U.S. Environmental Protection Agency (EPA) has identified phenol and its derivatives (2-chlorophenol, 2,4-dichlorophenol, and nitrophenol) as special pollutants due to their potent and hazardous properties [3]. These compounds are released into natural water reservoirs through industrial wastewater, posing serious health risks to humans and causing damage to the environment. It is crucial to reduce their presence in water, as they can cause significant damage and odor even at low levels of 5 μg/L [4]. However, the high stability and solubility of phenolic contaminants make their removal from aquatic systems quite challenging. Various methods of physio-chemical water treatment such as adsorption and photocatalysis are widely used to eradicate phenolic substances from water and wastewater. Adsorption is the most commonly used technique for removing phenol from wastewater due to its robustness, cost-effectiveness, and low energy requirement. Many adsorbents, such as activated carbon (AC), zeolites, and green nanomaterials, are being utilized for this purpose [5,6]. Photocatalysis has emerged as a promising green method for environmental remediation, utilizing non-hazardous light sources and non-toxic photocatalysts. Semiconductor nanoparticles, such as TiO_2_, V_2_O_5_/N, S-TiO_2_, BiPO_4_, ZnO, Al_2_O_3_, SiO_2_, and graphene-based materials, have been reported for the photocatalytic mitigation of phenolic pollutants [[7], [8], [9], [10], [11]]. Owing to the complex and non-linear nature of these processes, modeling and optimization are important for determining the optimal conditions of operation to reduce costs and time.

The surge of Artificial Intelligence (AI) has revolutionized the focus of scientists and researchers across the globe. AI is a field of computer science designed for resolving difficulties in a pattern similar to human intelligence. With the ultimate advancements in machine learning during early 1990s, there has been a lot of progress in its practical applications, leading to development of different AI techniques. Adding AI applications into a strategy seeks to enhance computer functions associated with human cognitive processes, including perception, logical thinking, learning and problem-solving [12]. Due to its remarkable efficiency, AI can perform classification and regression analysis of enormous quantities of data, stimulating progress in various fields. With advancements in super-computing abilities and the era of big data, AI has been successfully utilized in the medicinal, industrial, scientific, agricultural, and environmental sectors. The inclusion of AI in wastewater treatment has remarkably enhanced operational efficiency. This can be ascribed to AI deployment's small size, simple design, broad applicability and adaptability to changing circumstances [13]. Machine learning algorithms (MLAs) and AI can be easily integrated into intricate environmental systems to provide effective real-time defect detection, optimization, monitoring, and uncertainty prediction. The utilization of AI in water treatment allows for overcoming the shortcomings of conventional methods. In the present era, AI is receiving significant investments from water industries, with an expected reach of 6.3 billion dollars by 2030. AI is anticipated to reduce water treatment operational costs by 20–30 % [14] The resources and efforts can be saved by employing computational tools, such as AI, for prediction of performance efficiency.

The environmental contamination controls are characterized by different complexities such as multiple objectives and sources, sudden time changes and non-linearity. These complications pose difficulties in efficiently achieving significant system performance and optimizing important factors. Many statistical approaches such as k-means clustering algorithm (K-means), multiple linear regression (MLR), partial least squares regression (PLS), and response surface methodology (RSM) have been employed for dealing these issues.

The results of researches that present the contrast between AI models and statistical techniques revealed that former demonstrated eminent performance in terms of precision and accuracy across different tasks. However, the later have presented scientifically reliable knowledge based on statistical testing [15]. Therefore, unlike conventional empirical models, AI can precisely predict the eradication efficiency of phenolic compounds from water and elaborate on the relationship between operating conditions and removal ability. Artificial perceptron inspired from biological neuron is used to develop an AI model called Artificial Neural Network (ANN). These ANNs have been used for modeling and optimization of complex processes of different scientific domains. The self-learning and self-adapting abilities of ANNs make them desirable for this purpose. ANNs are being used for modeling complicated systems due to their robustness, simplicity, and non-linearity [16]. They mimic biological nervous processing and have a tendency to generate predictions in systems where there is insufficient knowledge about a given interaction. The ANN paradigm relies on identifying patterns in the data and differentiating between them to determine the pattern that leads to the desired conclusion. Intelligent backpropagation steers the process, enhancing the output model until the intended result is achieved. This methodology exhibits more precision and can effectively replace alternative modeling techniques. In order to enhance response prediction, a numerical search method that functions through processes similar to natural selection and genetics is needed to supplement ANN's capabilities [17]. ANN models frequently outperform conventional linear and non-linear models, according to scientific data [18,19]. ANNs uncover hidden relationships in historical data, aiding in the prediction of water quality [20].

A study using ANNs reported that optimizing process operations can cut material costs by 16.91 % and energy costs by 11.20 % in wastewater treatment, showcasing the potential of advanced technologies to enhance efficiency and sustainability [21]. An ANN accurately modeled fluid flow rates and pump energy consumption with less than 3 % error, optimizing sewage pumping systems for performance and efficiency while minimizing energy use. The results showed an average 10 % energy savings, with most significant achievement of 25 % reduction in energy utilization, exhibiting remarkable potential for energy conservation in sewage pumping operations [22]. ANNs can reduce the operational cost of treating phenol-contaminated water by modeling and anticipating optimal phenol removal conditions using datasets of operational factors [14,23,24]. The ANN optimization of Fenton reagents for treating phenolic effluents was carried out using Feed Forward Neural Network (FFNN) with ideal topology of 2:32:2. Before modeling, the efficiency of process was only 45 % but optimization of parameters increased it to 82 % saving 51.6 % of reagent costs [24].

**Fig. 1:**
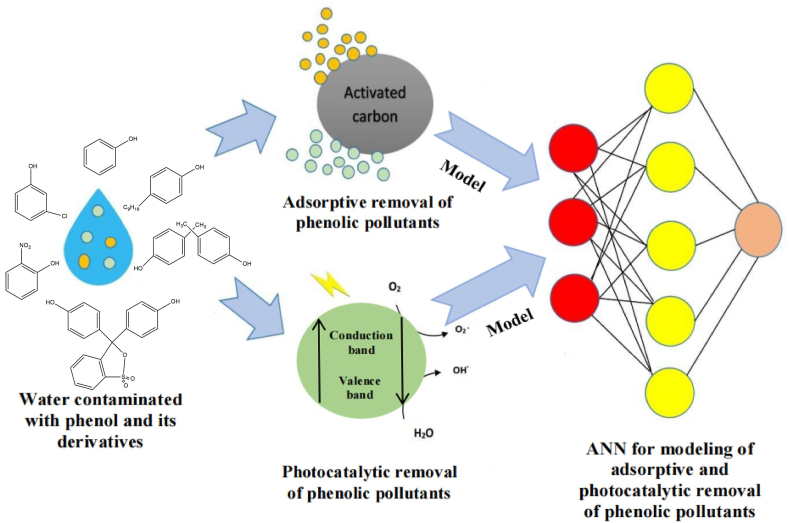
Illustration of adsorptive and photocatalytic mitigation of phenolic pollutants from water using ANNs.

From the last few decades, the ANN modeling of adsorption processes has drastically increased. The break-through, kinetics, thermodynamic and isothermal curves of a wide variety of adsorbates and adsorbents have been correlated and predicted using these AI models in the context of water purification. Artificial neural networks can overcome the major drawbacks of conventional adsorption models, particularly with regard to improving forecast performance across a range of operational scenarios [25]. Adsorption technologies can be deployed for phenolic pollution management and remediation, and researchers and policymakers can utilize ANN-enhanced adsorption models for demonstrating the impacts of adsorption processes on the environment. In multi-component adsorption systems, the use of ANN algorithms is a valuable technique for calculating isothermal, kinetic, and parameters [26]. Additionally, it can be utilized to model the non-linear relationship between photocatalytic parameters, such as phenolic pollutant size and concentration, presence of oxidants and electron acceptors, reaction temperature, catalytic loading, and their outputs, in order to aid in decision-making regarding the operation of a photocatalytic reactor [27]. ANN is capable of analyzing intricate correlations between variables such as reaction temperature, catalyst structure, and light intensity. More effective photocatalysts can be developed as a result of exposing latent patterns and impacts that conventional scientific approaches could overlook. In order to ensure that photocatalytic systems operate at their best, ANN can adjust reaction conditions in real-time. This results in lower expenses, more efficiency, and a smaller environmental impact [28]. The schematic illustration showing modeling of phenol removal has been shown in Fig. 1.

Researchers have been working to optimize the treatment of phenolic wastewater in an effort to save expenses, protect the environment, and shorten treatment times. Therefore, it is necessary to focus on such methods which offer eminent modeling of phenol removal and reduced limitations. Intending to model and optimize these processes, ANNs are most widely employed AI tools. They are emphasized owing to their parallel computing and self-regulation abilities, elimination of complex analytical and numerical iterative computations, handling of high dimensional data and uncovering hidden insights or patterns. The main goal is to develop method that is apt at predicting remarkable phenol removal efficiency and ANNs have practical applicability for this cause [29].

The objective of this review is to discuss the fundamentals and applications of ANN in treatment of water contaminated with phenol and summarizes recent studies on the modeling and optimization of the photocatalytic and adsorptive mitigation of phenols. Additionally, it provides a comprehensive discussion on the input and output parameters, the topology of the ANN model, degraded phenolic pollutants, percentage degradation, the adsorbent/catalyst used, the correlation coefficient, and the significant influential parameters affecting the efficiency of degradation. Further, this study compares the effectiveness of ANN modeling of phenol removal with other AI techniques, such as random forest (RF), adaptive neuro-fuzzy inference system (ANFIS), and support vector machines (SVM). To the best of our knowledge, there may hardly be a study in this area while critically reviewing the ANN modeling of phenol mitigation in detail, therefore, the current review addresses a huge gap of knowledge in the specified area. The most recent progress in this field has been cited. Various challenges and problems that hinder the employment of ANNs for the removal of phenolic pollutants have also been discussed.

### 1.1 Phenolic contaminants in water

The International Program on Chemical Safety suggests that the exposure to even minute quantities of untreated phenolic compounds (9–25 mg/L) can pose serious threat to human beings, animals and marine or freshwater aquatic life [30]. Various countries have enacted strict permissible limits of phenolic substances in different water quality standards for protection and sustainability of environment [31]. According to European Community, the concentration of phenolic contaminants should not exceed 1 mg/L in general wastewater [32]. The major contributing sources of phenols in wastewater involve degradation of aquatic plants and sewage discharge that is expelled during manufacture of pesticides and petrochemicals. Certain plants can synthesize phenol by exposure of chlorophyll to either UV radiations or chemical stress, such as effects of pesticides and infestation by microbes. Phenol is also produced as metabolic waste in bodies of human beings and animals during transformation of tyrosine in digestive system [33]. In addition to complex multicellular organisms, unicellular microbes such as *Debaryomyces hansenii,* can transform ferulic acid into various phenolic compounds, with the assistance of nitrogen and glucose [34]. This discharge has many deleterious and harmful effects either on soil and surface or ground water The European Community has allowed maximum limit of total phenol content in tap water to be 0.5 μg/L, and a permissible limit of 0.1 μg/L for individual phenols [35].

**Fig. 2:**
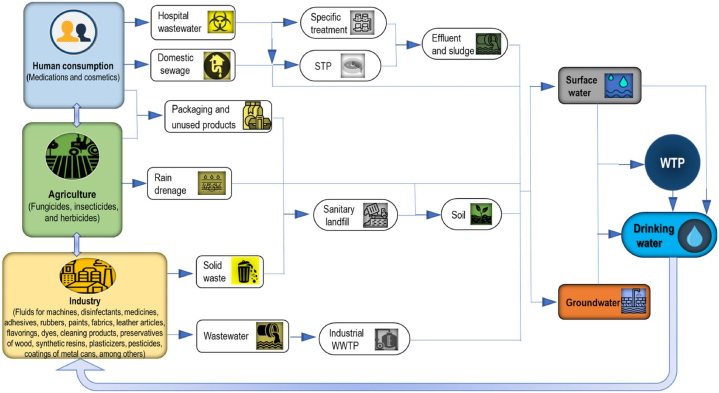
Anthropogenic sources of phenolic contaminants in water. WTP: Water Treatment Plant, WWTP: Wastewater Treatment Plant, STP: Sewage Treatment Plant. Reprinted from Ref. [50] with the permission of Elsevier.

Human beings are utilizing phenols in their daily life, either in petrochemical industries or manufacturing of chemicals. It was primarily employed as an intermediate for synthesis of chemicals including resins, cresols and alkylphenols [36]. To suit varied demands, phenolic resin was primarily employed in the wood sector, construction industries, and numerous appliances [37]. Moreover, the phenolic derivatives have been utilized in dyes, explosives and textiles industries as one of raw materials. Nitrophenols, a contagious class of environmental pollutants, are used in preparation of explosives, paper, textiles, pesticides, insecticides, plastics and pharmaceuticals. The isomers of nitrophenols exist in agricultural and sewage runoff owing to biological decay of organophosphorus pesticides and also in sea. Para nitrophenol is a toxic and carcinogenic chemical substance that is present in water samples as a consequence of industrial and agricultural activities [38]. Bisphenol A (BPA) is an emerging pollutant which is used in manufacture of epoxy and polycarbonate resins. These resins are employed for making coatings of infant milk bottles, water containers, and medical instruments. Three million tons of BPA are produced worldwide, annually. Due to its massive utilization, BPA is known to be present in all compartments of ecosystem i.e. air, water and soil. It is estimated that 92 % of BPA contamination occurs in water bodies due to which it would become an evident water contaminant in the future [39]. BPA occurs globally in ground water, surface water, rivers and seas. The inadequate wastewater treatment and leaching from BPA based micro-plastics causes BPA to enter aquatic habitats. The landfill leachates and effluents of municipal wastewater are major contributors of BPA contamination in groundwater and freshwater streams [40]. Chlorophenols (CP) are environmental pollutants arising due to chemical and pharmaceutical industry activities. Their existence is attributed to manufacture, utilization and decomposition of various pesticides including chlorobenzenes and chlorinated cyclohexanes. Most common of all, 2-chlorophenol is a liquid having unpleasant smell which is mainly used in preparation of disinfectants, germicides and dyes [41]. According to WHO, 4-CP is also classified as a toxic and carcinogenic waste, known for contaminating soil and water bodies, due to its bio-accumulation in food chain and harmfulness towards environment [42]. Alkylphenols are abundant environmental pollutants that are continuously introduced into environment from natural sources and human activities. Cresols are produced on a massive scale with global annual production surpassing 470 kilotons because they are employed in manufacturing of pesticides and plasticizers. Alkylphenols are associated with crude oil and coal tars due to which coal refineries discharge them in large amounts during gasification. Alkylphenols have been found in landfills, groundwater and water near industries in amounts ranging from micro-to-nanomoles [43]. The industrial processes which are concerned with manufacture and/or utilization of phenolic compounds are responsible for their discharge into the ecosystem and in contamination of ground or surface water bodies. Remarkable phenolic compound concentrations are present in wastewaters from oil industries or refineries, pharmaceutical industries and tanneries. In several wastewaters such as olive mill wastewater, the most toxic class of pollutants is phenolic compounds [44]. Environmental Protection Agency (EPA) regulations require nitrophenol concentration in water to be less than 10 ng/L [45]. The guide on quality of drinking water (USEPA) recommends a maximum concentration of 28 μg/L for nonylphenol in fresh water [46]. Both in the second and third edition (1993 and 2003) of the Guidelines for Drinking Water Quality (WHO), the provisional guideline value of pentachlorophenol in drinking water was 9 μg/L. EPA's Maximum Contaminant Level for PCP in drinking water was 1 μg/L [47]. The WHO takes phenolic compounds as harmful with a maximum admissible concentration in drinking water of 200 μg/L for 2,4,6-trichlorophenol and 10 μg/L for 2-chlorophenol [48] and the total allowable concentration of 0.1 mg/L is recommended by The National Sanitation Foundation for BPA [49]. Various anthropogenic sources of phenolic contaminants in water and treatment plants have been provided in Fig. 2.

### 1.2 Harmful effects of phenolic contaminants

The existence of phenolic contaminants is a common problem among the global population due to natural and chemical processes taking place in industries. The structure of phenol itself is responsible for its toxic traits including sustenance in environment and possibilities of carcinogenic characteristics toward living beings [51]. Owing to deleterious effects of these pollutants on human health and toxicity, persistent nature and bio-accumulation inside bodies of animal, plants and human beings, the presence of phenols in sewage treatment plants has emerged as an alarming issue. Unfortunately, many untreated phenols in effluents are substantially dangerous for aquatic ecosystems. A chemical compound's toxicity to a living being is often determined by looking at its median lethal concentration (LC_50_) and median effect concentration (EC_50_). According to Verma et al., *Cirrhinus mrigala* had a 96-h lower limit of sensitivity (LC50) of 1.555 ppm of phenol, making it the most sensitive freshwater organism [52]. Freshwater fish, *Oncorhynchus mykiss*, had a fifty percent higher death rate after four days of exposure to 3.88 ppm m-cresol [53]. The freshwater-dwelling water flea, *Daphnia magna* exhibited the highest sensitivity to 5.0 ppm of o-cresol and 1.4 ppm of p-cresol with a 48-h linear correlation coefficient [54]. Among the organisms dwelling marine ecosystem, *Archaeomysis kokuboi* exhibited greatest phenol susceptibility, with 96 h LC_50_ of 0.26 ppm and the highest sensitivity towards p-cresol is shown by *Oncorhynchus gorbuscha* with 96 h-LC_50_ of 3.36 ppm [55]. The rotifer *Philodina acuticornis* was shown sensitive towards 325–371 ppm *of* phenol with 24 h-LC_50_ and *Brachionus calyciflorus* exhibited mortality towards 111.5 ppm of phenol with 24 h-LC_50_ [56,57]. The molluscs including *Unio tumidus, Anodonta piscinalis, Dreissena polymorpha* exhibited mortality towards 1000 ppm of phenol with 48 h-LC_50_ [58]. The crustaceans such as *Carcinus maenas, Gammarus pulex, Gammarus minus, Macrobrachium rosenbergii and Palaemonetes pugio* exhibited mortality towards 56 ppm, 55–89 ppm, 37.4 ppm, 22.25–29.04 ppm and 11 ppm of phenol with 48 h-LC_50_ [[59], [60], [61], [62], [63]]. Acute and sub-chronic exposure to 100–200 mg/m^3^ of phenol caused harm and damage to kidneys, lungs, liver and heart of rabbits and guinea pigs; while in rats, this exposure lead to hepatic damage and impaired neurological functions [64]. Food containing naturally occurring sesamol, caffeic acid and catechol induced papillomas, proliferation and carcinomas in the forestomach of male F344 mice as significantly as synthetic polyphenolic antioxidants [65]. Chlorophenols are dangerous endocrine disruptors that can severely harm the central nervous system, endocrine, immune and reproductive system of animals. They can cause feminization of zebra fish [66]. The tendency of chlorophenols to induce carcinogenesis in liver of mice was elaborated by Umemura et al. [67]. Ucisik et al. showed how *Salix viminalis*, a willow tree, removes, absorbs, accumulates, and becomes poisonous when exposed to phenol. It was found that inhibition of transpiration began on exposure to 250 ppm of phenol. The rate of transpiration decreased by 50 percent at 500 ppm of phenol, and the trees wilted. However, the trees exposed to 1000 ppm of phenol wilted, died and finally decomposed [68]. It was found that metabolic and gut microbiome alterations occurred in dogs that consumed canned food having BPA for 14 days because 81 % of dog food cans have BPA based coating [69].

The phenolic compounds that exist in environment can enter human body through ingestion and dermal contacts. Most probably phenols present in water enter bodies of human beings through drinking water; while, the sedimentary phenols enter through food web. Owing to their persistent and non-biodegradable features, phenols can accumulate in water or sediment where marine and freshwater organisms dwell. When the exposure level fall outside the normal range or significantly change over time, then it may pose serious threat to aquatic life and human health [70]. Phenolic Organic Contaminants (POCs) are extremely harmful pollutants that find their way into human body through respiratory tract, digestive tract, skin, mucosa and chemical reaction with proteins present in body. The proteins undergo coagulation and denaturation, leading to inactivation of cell. POCs have strong penetrating power due to which they enter tissues, cause systemic toxicity in animals and humans and ultimately, lead to death in severe cases [71]. POCs are endocrine disruptors (EDCs) which harmfully affect the human organs. BPA and alkylphenols have been recognized to induce endocrine disrupting effects on human beings by changing the development of mammary glands in exposed animals [72]. BPA has capability to delay onset of puberty in females [73]. Relative to endocrine system, POCs can interfere with synthesis of important hormones due to which growth and development of human beings can be retarded. To some extent, these substances can lead to dysfunction of immune system, causing immune regulation disorder. This disorder can damage reproductive system of human beings leading to infertility. POCs can cause neurological disabilities such as inattention and memory loss [74]. These compounds hinder the preparation of DNA in diploid human fibroblasts, that proved their inhibitory effect towards DNA synthesis and replication in cells. Moreover, phenols can lead to rupture of internal membranous structure of a cell after penetrating into it. The drinking water containing high levels of phenols leads to gastrointestinal ailments and muscle tremor with trouble in walking [75]. The skincare products having high phenolic levels result in blisters and burns on skin. The chlorophenol (CP) poisoning is responsible for mouth and throat burning. The CPs are known to cause necrotic lesions in esophagus, mouth and stomach. They are also related to convulsions, fatigued muscles, irregular pulse and abnormal temperature [76,77]. Catechols can readily oxidize to quinones which are responsible for DNA arylation and damage. They can interrupt electron movement in energy transducing membranes [78]. DNA damage is also caused by caffeic acid and dihydrocaffeic acids in presence of Cu [79]. Various ailments associated with exposure to different kinds of phenolic pollutants have been listed in Table 1.

**Table 1:** Different diseases associated with exposure to different types of phenolic pollutants.

**Sr. No.**	**Phenol**	**Disease**	**Mechanism**	**Ref.**
1	2,4 Dichlorophenol 2,4,6 Trichlorophenol Pentachlorophenol	Thyroid Cancer	Disrupting the endocrine functions by altering hormone levels, inducing abnormal gene expression, stimulating cell proliferation, increase in DNA lesions and point mutations.	[80]
2	Urinary 2,5-dichlorophenol	Heart diseases and cancers	The mechanisms elaborating connections between 2,5-DCP and cardiovascular diseases and cancer are not yet identified.	[81]
3	Chlorophenols	Nasopharyngeal squamous cell carcinoma	Cytochrome P450 is microsomally activated upon exposure to chlorophenols. Oxidation causes xenobiotics to change into electrophilic structures that communicate with cells directly.	[82]
4	Nonylphenol	Ovarian cancer	Cell growth stimulation in ovarian cancer cells	[83]
5	4-Nonylphenol	Breast cancer	MCF-7 and T47D cell proliferation was stimulated.	[84]
6	4-Nonylphenol	Mammary cancer	Nonyl phenol significantly impacted the T47D mammary ductal carcinoma cell line's ability to develop.	[85]
7	Bisphenol A (BPA)	Ovarian cancer	inter cellular proliferation of OVCAR-3 cells.	[86]
8	BPA	Colon cancer	HT-29 human colon cancer cell proliferation and migration, phosphorylation of extracellular signal-regulated kinase (EKR), increased 5-HT3 expression, reduced E-cadherin expression	[87]
9	BPA	Prostate cancer	Stimulate cell proliferation and migration through AR-T877A, changes cell morphology, induces cell cycle arrest, changes methylation of anti-oncogenes, upregulation of p21 and p27, downregulation of EKR and cyclin D	[88]
10	BPA	Osteosarcoma	Inhibition of CDC42 expression, downregulation of OPG, RUNX2, COL1A1, interaction with LOX gene	[89]
11	BPA	Male germ cell cancer	Upregulation of PKG, EGFR/ERK/c-Fos and activation of GPR30, ERK,EGFR located at 5′- regions of GPR30	[90]
12	BPA	Acute myeloid leukemia	Upregulation of FAS, TRAIL and down regulation of Cyclin D1, IL-4	[91]
13	4-Nitrophenol	Bladder cancer	Increased expression of HIF-1, K-Ras, Decreased expression of BRCA, p53, PTEN	[92]
14	4-Nitrophenol	Colorectal adenocarcinoma, Burkitt's lymphoma	Induced growth of HT-29 cells (Colorectal adenocarcinoma) and Ramos.2G6.4C10 cells (Burkitt's lymphoma)	[93]

### 1.3 Various AI methodologies utilized for mitigation of phenolic contaminants from water

The treatment of wastewater, a complex process, generates a large amount of data using online sensors, paving the path for utilization of AI in systems for efficient and better performance. It is estimated that AI standout as a promising technique for data analysis in comparison to traditional statistical methods. The modeling capabilities of AI methodologies are very useful in wastewater treatment processes because of their robust and inexpensive operations [94]. Various AI models for simulation, prediction and improvement of mitigation of phenolic contaminants from wastewater have been extensively utilized. AI involves different methods, each with its benefits, drawbacks, and uses. ANN is distinguished for its effectiveness in handling large datasets and its ability to make predictions quickly. They can approximate any arbitrary function and are skilled at predicting sequences, such as forecasting time series events. Sometimes ANN methods, such as recurrent neural network, fuzzy neural network, convoluted neural network and deep neural network are computationally costly and need a lot of training data. Understanding and applying their interpretability is usually difficult, requiring specialized knowledge [95,96]. Other important AI methods for handling regression analysis and classification are support vector machines (SVMs) and support vector regression (10.13039/100019572SVR). The labeling of training data for every class set is necessary for the recognition of the following step. The mapping of an input vector into a high-dimensional space utilizing radial, polynomial, and linear basis functions is the fundamental idea behind the SVM's operation. In order to indicate the distinction between two classes, a hyperplane is drawn to widen the space between them [97]. The SVM has more mathematical theory backing than the traditional ANN, which improves the model's interpretability to some extent. Moreover, the SVM may be converted into a convex optimization problem, guaranteeing the algorithm's global optimality and avoiding the local minimum problem, which poses a challenge for neural networks to tackle [98]. However, these models need a lengthy training period and are challenging to train using large-scale sample data. Computationally demanding, particularly for extensive data sets, is a result of the necessity for quadratic programming during the training phase. Moreover, choosing the right kernel and adjusting the hyperparameters are important and difficult tasks that demand a lot of skill and knowledge. SVM models are not as easily interpretable as simpler models like ANNs, and their results are often not as easily understood [99,100]. A support vector regression model was applied for adsorptive removal of phenol using rotating packed bed as adsorbent where the coefficient of determination (R^2^) was found to be 0.996 [101]. It was also used for prediction of phenolic contaminants in drinking water by establishing relationship between regression equations of UV–Visible spectra of phenolic compounds and their concentrations [102]. Dragonfly support SVM model for predicting adsorption of various phenol on Activated carbon fibers was built that gave more accurate values corresponding to R^2^ = 0.997 [103]. SVM integrated with Bayesian Optimization (BO-SVM) was utilized as a predictive tool for mitigation of BPA using polyethyleneimine/graphene oxide/layered triple hydroxide nanocomposite as an adsorbent. The efficiency for BPA removal data was evaluated to be 96.6 % [104].

Decision Tree (DT) is an AI technique that predicts a category by “learning simple decision rules”. A tree like structure with edges representing possible solutions of problems, leaf nodes giving actual output or class label and nodes denoting characteristics of the data is included in this technique. In DT, the attributes of a problem are screened from root to leaf node for provision of optimal result based on conditions. DT was used for predicting the BPA concentration in 12 municipal Wastewater Treatment Plants (WWTP) with R^2^ = 0.859 [105]. It was utilized for predicting odor characteristics of sludge from WWTP that contained p-xylene and p-cresol [106]. Unfortunately, this technique has limited applicability owing to its instability and over-fitting. Random forest generates DT on data samples, makes an anticipation on each DT and finally, solves the problem with voting mechanism. It eliminates the demerit of over-fitting by taking average of the results. More the DTs, more accurate will be the RF [107]. Random Forest has some limitations. First, it might be computationally expensive, particularly for large trees and features. Secondly, it can lengthen the training period. Comparing the models produced by RF to simpler patterns like ANNs, the former can be more challenging to understand and handle [108]. It was used to investigate efficiency of phenol removal in backlight cascade photocatalytic reaction with TiO_2_ as photocatalyst with R^2^ = 0.9972 [109]. It was applied in investigation of sorption of chlorophenols on MWCNTs with the implementation of density functional theory. This model elaborated and validated the intermolecular interactions between phenols and eluents [110]. A RF model for adsorptive removal of bromophenol blue had been built using the activated carbon obtained from *Astragalus bisulcatus* tree with R^2^ = 0.9895 [111].

ANFIS is based on construction of layers of fuzzy if-then rules with proper membership functions for generating stipulated input-output pairs. The output of ANFIS is sometimes not precise and it also needs high-level expertise to convert natural language into Machine Learning language [112]. ANFIS model was built for predicting eradication of 2,4-dichlorophenol using activated carbon with R^2^ = 0.999 [113]. It was modeled onto persulfate/nano zero valent iron process for eradication of 4-chlorophenol from aqueous solutions with R^2^ = 0.9942 [114]. Another ANFIS model was utilized for computing the photodegradation % of 2,4-dichlorophenol that is broken down by Fe_3_O_4_/TiO_2_/Ag photocatalyst [114]. It was used for predicting 4-chlorophenol degradation using persulfate, oxalic acid and heterogeneous Fenton like system with high accuracy (R^2^ = 0.98) [115]. The ANFIS model for predicting photocatalytic performance of SnO_2_/Fe_3_O_4_ nanoparticles for degradation of phenolic dyes was also built that exhibited R^2^ = 0.9023 [116]. Genetic algorithm (GA) is an AI technique that treats population of possible solutions in a similar manner to biological genetics and selection. The possible solutions are encoded as genes comprising of characters in form of an alphabetical string. A mutation from rest of the population members generates new solutions, and ultimately, via mating, two solutions merge to give rise to a new one. This methodology is also computationally expensive, function of fitness is not clear and selection of best solution is difficult [117]. It was used to investigate magnetic chitosan's adsorption capacity towards phenol from synthetic solutions and it was found that maximum removal efficiency was 97 % [118]. It was employed in adsorption studies of phenol mitigation using banana and grapefruit peels [119]. It was applied in estimation of phenol degradation by bacteria of *Alcaligenes, Providencia, Acinetobacter* and *Aeromonas* genera. The higher values of obtained kinetic parameters showed that these bacterial strains were able to withstand and mitigate higher phenol concentrations [120]. k-Nearest Neighbor is simple machine learning technique that saves all the existing information and classify it on basis of new data points in accordance to similarities. Its main drawbacks include the computational expense of distance calculations and memory intensity [121]. It was utilized along with Gray Wolf Optimizer for building a predictive model to estimate adsorptive mitigation of phenol on powdered AC. This model predicted the amount of phenol adsorption with higher statistical coefficient i.e., R^2^ = 0.9998 [122]. Two ANN models (GRA–CNN–LSTM model and PCA-BPNN model) were trained by a large data for the prediction of quality and quantity of wastewater. The overall average prediction accuracy of 92.60 % and 93.76 % were obtained for inlets/outlets wastewater indicators and on combining these models more than 11.20 % reduction of energy was obtained [21]. A small-scale BWRO plant was developed by employing ANN and RSM methodology. The results showed an energy consumption of 17.60 kWh/m^3^ and it was revealed that plant has significantly minimized the energy consumption [123].

### 1.4 Artificial neural networks (ANNs)

**Fig. 3:**
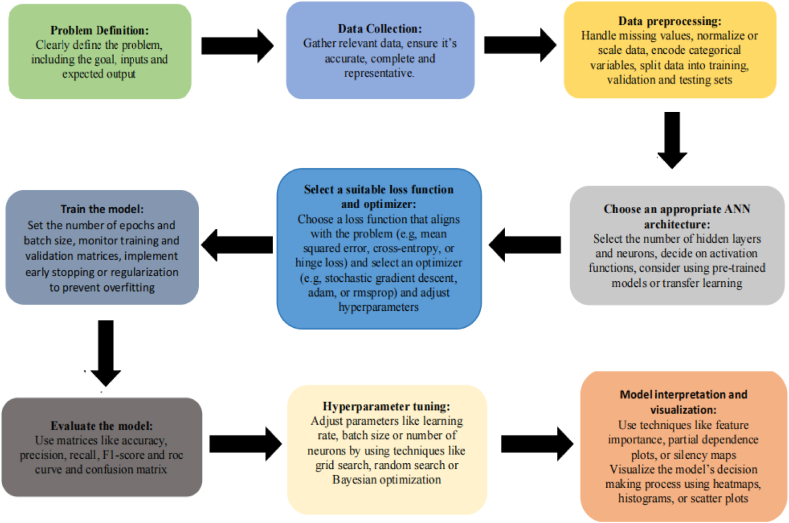
General guidelines to start with the ANN modeling.

ANN is a computer model inspired by brain function and structure. Artificial neural networks consist of nodes called neurons that are interconnected and organized together. In general, a neural network with an input layer, an output layer, and one or more hidden layers is combined to form an ANN. In the functioning of an Artificial Neural Network (ANN), a series of distinct steps come into play. The general guidelines to start with ANN modeling have been shown in Fig. 3.

It all begins with the input layer, which receives the initial data or information and then transmits it to the subsequent layer, called hidden layer where hidden layer is a collection of neurons that transmit the training and data between the layers Each neuron in the hidden layers and the output layer calculates the weighted sum of the inputs it receives from the previous layer and processes it through an activation function, such as the widely used ReLU function. The resulting output is then forwarded as the input to the next layer, and this process continues until the final output is obtained from the output layer [124,125]. A high number of hidden layers are favorable for accuracy but not for time complexity. During the training phase, the ANN fine-tunes the weights of the connections between neurons based on the disparity between the expected and actual output. This adjustment is typically achieved using backpropagation, which involves updating the weights through optimization algorithms like gradient descent and calculating the error gradient with respect to the weights [126]. This iterative process enables the ANN to learn and adapt, ultimately improving its ability to make accurate predictions or classifications based on the input data and hence, prior to the designing of ANNs it is needed to analyze the training database samples [127]. The efficacy of the model of the artificial neural networks can be measured by the parameters such as Mean Absolute Error (MAE), Mean Squared Error (MSE), Root Mean Squared Error (RMSE), Mean Absolute Percentage Error (MAPE), Adjusted R^2^, Precision, Recall, and F1-Score, Confusion Matrix, Receiver Operating Characteristic Curve (ROC) and Area Under the Curve (AUC) etc. By including a selection of these metrics, a more comprehensive evaluation of neural network's performance can be provided. The specific choice of metrics may depend on the nature of the problem (e.g., regression vs. classification) and the specific goals of analysis [128].

## 2 Significance of ANN for the removal of phenolic contaminants

### 2.1 Adsorption

Among the popular technologies for treating water, adsorption is thought to be a safe, affordable, and effective way to remove phenolic contaminants from water. The process offers a versatile design and operation that can generate treated wastewater devoid of odor, colour and sludge. The reusable and regenerable adsorbent makes adsorption an appealing and cost-effective method. It is also intriguing that adsorption doesn't result in secondary waste because, even in the case of diluted solutions, no sludge is produced during the phenol removal process. Moreover, adsorption may remove contaminants at low concentrations while using little energy [129]. Adsorption is the process by which molecules of a gas or liquid (absorbate) adhere to the surface of a solid or liquid (adsorbent) to form an atomic or molecular film. This occurs as a result of residual or imbalanced forces on a liquid or solid phase's surface [130]. As the molecular species approach the surface, the residual imbalance forces keep them drawn in and hold them in place. Owing to the bonding forces like covalent bonds, which are strong forces, or van der Waals forces, which are weak forces, the adsorbate is absorbed by the adsorbent [131]. Adsorption is an interactive process that, depending on the intermolecular forces, binds the liquid phase component to the surface of the solid adsorbent either physically or chemically. This method of isolating the desired contaminant from the aqueous solution is also known as segregation, and it can be done through continuous, semi-batch or batch experimentation [132].

**Fig. 4:**
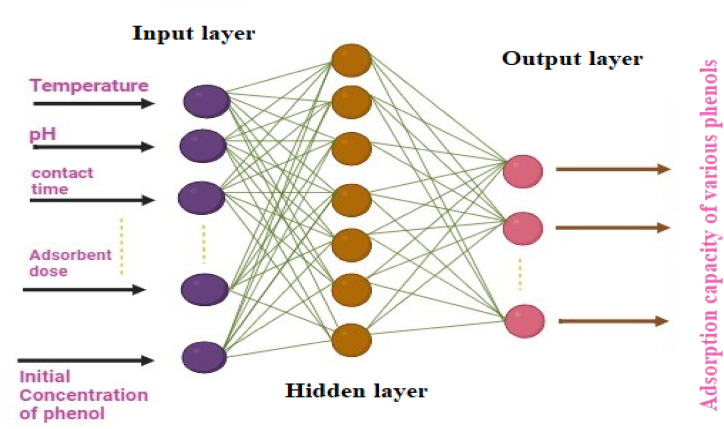
ANN model for adsorptive removal of phenol from water.

Adsorption can be divided into two types: physical and chemical adsorption. Physical adsorption occurs when weak forces, such as dipole-dipole interactions, hydrogen bonding, van der Waals forces, and polarity exist between the adsorbate and adsorbent. Physiosorption takes place at temperatures comparable to or slightly lower than those of the adsorbed components [133]. Chemical adsorption, on the other hand, is the interaction of the adsorbate with the adsorbent's surface via chemical bonding or electron transfer. This type of adsorption, also known as activated adsorption, is distinguished by a permanent reaction that requires a large activation energy. Chemical adsorption, unlike physiosorption, is irreversible [134]. A multilayer adsorption process with a large adsorption capacity can take place via physical adsorption. Conversely, chemical adsorption can only remove specific trace substances from aqueous solutions and is restricted to monolayer adsorption. Because of this irreversible reaction, the adsorbent's regeneration and reusability are especially challenging for chemical adsorption [135]. Adsorbate diffuses on the adsorbent's outer surface as a result of the diffusion potential, which is established by the adsorbent's accessible outer surface area and the adsorbate concentration. Diffusion potential can occur in a single step or in a series of phases, including adsorption on the pore surface, film or external diffusion, surface diffusion and pore diffusion. The adsorbate is then dispersed across the adsorbent's available pores. All exposed active sites are filled with either chemical or physical adsorption during the adsorption process [136]. Artificial Neural Networks (ANNs) play a crucial role in the modeling adsorptive mitigation of phenolic contaminants from water. By utilizing ANNs, the adsorption of phenolic contaminants from wastewater can be optimized, leading to more efficient removal. ANNs can aid in predicting the maximum uptake of different adsorbents, enabling the selection of the most effective materials for the removal process. Additionally, ANNs can assist in modeling the adsorption kinetics and isotherms, providing valuable insights for the isolation of the adsorbent from the treated water. The application of ANNs in this situation may ultimately aid in the creation of more long-lasting and efficient methods of treating water to remove phenolic pollutants. The general ANN modeling of adsorptive removal of different phenols has been shown in Fig. 4.

Ghaedi et al. reported the adsorptive eradication of phenol red using TiO_2_ and gold nanoparticles incorporated on AC. The Neural Network MATLAB 7.12.0 (R2011a) mathematical software was used to predict removal percentage. For modeling and optimization of adsorbents i.e., TiO_2_-NP-AC and Au-NP-AC removal potential, a three layer ANN model was used. This model trained with Levenberg Marquardt backpropagation (LMBP) had purelin at output layer and tansig at hidden layer. The input parameters were contact time (1–36 min), pH (1–8), [phenol red]_0_ (2–200 ppm) and mass of adsorbent (0.005–0.035 g) while, the mitigation (%) was output parameter. The number of neurons in hidden layer were varied from 1 to 23. For TiO_2_-NP-AC, the optimum number of hidden layer neurons was 19 that lead to achievement of MSE and R^2^ values of 0.0000566 and 0.9994, respectively. ANN with 15 hidden neurons allowed to obtain MSE and R^2^ values of 0.0022 and 0.9729, respectively for Au-NP-AC. The maximum adsorption occurred at pH = 3 while, the lowest adsorption occurred at pH = 2. By increasing adsorbent mass from 0.005 to 0.035 g, the removal efficiency increased. The rate of adsorption was higher in the initial 30 min of adsorption. By increasing [Phenol red]_0_ from 60 to 120 ppm, the amount of phenol red adsorbed increased from 54.6 to 73.2 mg/g for TiO_2_-NP-AC and 49.2–62.4 mg/g for Au-NP-AC [137].

Another LMBP trained model based on multilayer perception method (4:6:1 configuration) was employed for phenol adsorptive eradication model using NiFe_2_O_4_ nanocomposite as adsorbent. The input parameters were concentration of NiFe_2_O_4_ composites, pH, initial amount of phenol and contact time. Out of 31 experimental data sets, 23 were used in training, 3 were tested and 5 validated. The model predicted 99.83 % of phenol mitigation with 0.15g of NiFe_2_O_4_ composites after a contact period of 59.97 min and pH = 7.67 for 128.68 mg/L of phenol concentration. The R^2^-values for testing, validation and training were 0.9784, 0.9652 and 0.9934, respectively; and as all of these values are close to 1 thus, the relationship between mitigation efficiency and input parameters is satisfactory [138]. In another study, the sorption of 3-amino-phenol and phenol was investigated on Fe nanocomposite adsorbent using ANN model with 5:8:1 configuration. The input parameters were concentration of composite, pH, contact time and initial concentration of contaminant; while the dependent variable was uptake effectiveness. Maximum uptake efficiency of sorbent was 85 % for phenol and 80 % for p amino phenol. Model was used for predicting the relative error in sorption efficiency that were in the range of 0.35–3.5 for p amino phenol and ±0.35 to 3.0 for phenol [139]. Multilayer perception (MLP)-ANN was applied for estimation and validation of BPA adsorptive removal using zeolite imidazole frameworks (ZIFs). The porosity of adsorbent was very high owing to its pore volume and specific Bruner-Emmett-Teller (BET) area that were 0.60 m^3^/g and 1299 m^2^/g, respectively. The input parameters were pH, contact time, BPA concentration and adsorbent (ZIFs) amount. Out of 116 data sets obtained from input parameters, 70 % were trained and 30 % validated. The adsorption efficiency was found to be 99.93 %. The ideal ANN topology (4:10:1) gave R^2^-values of 0.983 and 0.9704 for validation and training, respectively. The RMSE values for validation and training were 0.5252 and 0.2214 [140]. Another study reported the phenolic waste mitigation using fine-grained soils. A 3 layered FFNN was employed that had purelin at output layer and tansig activation function at hidden layer. The model was trained using LMBP algorithm was developed using MATLAB Neural Network Toolbox version 8.0. The input parameters included contact time, adsorbent dosage, % of clay, % of silt and [phenol]_0_ while, phenol removal % was output parameter. Out of 460 data sets, 320 were used for training, 160 were validated and 160 tested. The number of hidden nodes was varied from 2 to 34. The favorable number of hidden layer neurons was selected as 26 because the MSE value was 0.006. The ideal ANN topology was 5:26:1 with R^2^ of 0.993 [141].

A novel multiwalled carbon nanotubes (MWCNTs) adsorbent functionalized with deep eutectic solvents (DESs) for mitigation of 2,4-dichlorophenol from water was prepared. The adsorption effectiveness was predicted by FFNN trained with LM backpropagation. The input parameters were pH, 2,4-dichlorophenol concentration, MWCNT dosage and contact time, while the output variable was adsorption efficiency. Out of 147 experimental data sets, 25 were utilized in testing and 122 were used in training and validation. The designed model had 1 input layer with four neurons, 2 hidden layers with 10 neurons in each layer and one output layer. This model was developed using MATLAB R2014a. The ideal ANN topography contained 10 neurons in both hidden layers, with mean square error (MSE) equal to 0.00005 and R^2^ = 0.9993. These values were in agreement to those obtained from experimental data [142]. Fe_3_O_4_/AC NPs were prepared for adsorptive mitigation of nonylphenol from contaminated water. The ANN model was used for elaborating influence of input parameters on eradication of nonylphenol. The input parameters included [Nonylphenol]_0_, pH, time and adsorbent dosage while the nonylphenol removal efficiency was output variable. The number of hidden neurons were varied from 2 to 20 for obtaining most appropriate ANN topology. The MSE values of training and validation sets determined the optimum number of hidden neurons. The ideal ANN topology was 4:12:1 with R^2^ of 0.986. It was found that nonylphenol removal occurred quickly at 30 min of reaction time and pH of 3. The increase in initial concentration of nonylphenol and adsorbent dosage also increased the adsorption efficiency. The adsorption efficiency decreased after 5th cycle from 99.6 to 92.6 %. The adsorption efficiencies for wastewater effluent, tap water and river water were found to be 67.3, 73.5 and 70.7 %, respectively [143].

Shahryari et al. utilized a typical 3-layered FFNN trained with LM backpropagation for modeling adsorptive removal of phenol using AC. The input parameters were pH, temperature, contact time, adsorbent and phenol concentration. Out of 102 data sets, one half of data sets (50) were utilized for training, one-fourth (26) were used in validation and one-fourth (26) were tested. The number of neurons in hidden layer were altered from 2 to 11. The smallest mean square error value of 0.0565 was found when the number of hidden neurons was 9. The ideal ANN topology had configuration 5:9:1. This model was developed using MATLAB. The adsorption percentage of 99 % was achieved at natural pH, 290 K temperature and 100 mg adsorbent dosage. The proposed ANN model predicted phenol adsorptive removal with R^2^ and RMSE of 0.9998 and 0.2378, respectively. When number of neurons was small then they were not able to understand input and output parameters efficiently but with high number of neurons, structural complications like computational errors and increased training time were observed [144]. The mitigation of BPA from aqueous medium using fixed-bed column of cross-linked chitosan/zeolite (CCZ) was reported. The removal efficiency of CCZ for BPA was optimized using ANN and RSM. ANN modeling of adsorption was carried out employing the input parameters included [BPA] and pH while, the output parameter was BPA eradication. This ANN model was developed using MATLAB R2015a. The favorable number of hidden layer neurons was four owing to lowest MSE and highest R^2^. The LM training began after 23 epochs to reveal that ANN model was trained appropriately at the end. 60 % of data sets were used for training, 20 % were validated and 20 % tested. The maximum eradication of BPA was 92.9 % that occurred at [BPA]_0_ = 1.70 ppm and pH = 5 while, the lowest removal of BPA was 47.3 % that occurred at [BPA]_0_ = 2 ppm and pH = 8. The R^2^ values of training, validation and testing for removal of BPA were 0.998, 0.998 and 0.997, respectively. The MSE values of training, validation and testing for removal of BPA were 4.62, 6.91 and 1.08, respectively. However, RSM revealed 89.0 % removal of BPA at pH = 5 and [BPA]_0_ = 1.456 ppm, with R^2^ of 0.997 [145].

A study was carried out for mitigation of BPA using Hydroxyapatite/NaP zeolite. ANN modeling was done for estimation of adsorption efficiency over fabricated adsorbent using data obtained from batch experiments. A 3 layered FFNN-MLP was used for predicting synthesis of HAp:zeolite, availability of surface area and total adsorption. The input parameters were temperature (298–318 K), pH (5–12), [BPA]_0_ (5–50) mg/L, time (30–120 min), adsorbent dosage (1–4 g/L) while, the output parameter was % Removal BPA. Out of 70 data sets, 48 were trained, 11 were validated and 11 tested. The number of hidden layer neurons were varied from 2 to 25 for and the optimum number was selected as 12 owing to minimum MSE. The ideal ANN topology was 5:12:1 with R^2^ of 0.9905 and MSE of 0.005938 [146]. ANNs have been used for modeling the adsorption of phenol onto barley husks AC. The input parameters included adsorbent dosage, flow rate of air, influent flow rate and influent concentration. Out of 87 data sets, 70 % were used for training, 15 % were validated and 15 % tested [147].

Sharafi et al. reported the adsorptive mitigation of phenol from aqueous solutions using scoria stones. The adsorbent was modified by treating it with HNO_3_, HCl, H_2_SO_4_, CH_3_COOH and H_3_PO_4_ in order to enhance porosity, surface charge and sorption capability. The removal efficiency of modified adsorbents was compared using RSM and ANN. A 3 layered FFNN was used for modeling of process and the topology was determined by merging ANNs with Clonal Selection (CS) and the topology was determined by merging ANNs with Clonal Selection (CS). The role of CS was to optimize ANN model of adsorption process and determination of the influence of variables leading to maximum removal. The input parameters were contact time and adsorbent dosage while, the output parameter was mitigation of phenol. The ideal ANN topology was 3:10:1. The concentration of phenol adsorption on acid treated scoria was found in decreasing order of: H_2_SO_4_> HNO_3_ >CH_3_COOH > H_3_PO_4_ >HCL. It was found that there is no 100 % compatibility among experimental data sets and models. For instance, in case of untreated scoria, the experimental optimum was 89.12, while maximum predicted by CS-NN was 90.2 and by RMS was 100. In fact, for all cases, RSM optimization gave solution with 100 % mitigation of phenols, while CS-NN was more closer to experimental points [148]. The removal of 4-CP was studied using dried anaerobic digested sludge (DADS). ANN model developed using MATLAB R2015a was applied for predicting efficiency of removal. A 3 layered FFNN with tansig at hidden layer and purelin at output layer was utilized. 70 % of data sets were trained, 15 % were validated and 15 % tested. The input parameters were contact time, pH, DADS dosage and [4-CP]_0_ while, the output parameter was removal percentage. The number of hidden layer neurons were varied from 1 to 20 and the optimum number of hidden nodes was selected as 10, with lowest MSE of 0.000841. The ideal ANN topology was 4:10:1 with R^2^ of 0.98. The optimum pH for adsorbent was 3. The removal efficiency increased with DADS dosage (96.6 % with 30 g/L of DADS) and contact time but it decreased with rise in initial concentration. These findings elaborated that results predicted by ANN are in accordance with experimental values [149].

Three ANN models were employed for eradication of phenol and resorcinol from water. They utilized traditional and inexpensive adsorbents including activated carbon (AC), wood charcoal (WC) and rice husk ash (RHA) for designing model A, B and C. Out of 29 data sets, 15 were trained, 7 were tested and 7 validated. The input parameters were concentration of adsorbent, prior amount of phenol and resorcinol, pH and contact time while, the output variable was mitigation efficiency against phenol and resorcinol. The ideal ANN architecture for all the models was 5:8:1. These ANN model were designed using MATLAB Neural Network Toolbox version 7. The performance of models was evaluated using statistical parameters such as mean square error (MAE), coefficient of determination, root mean square error and mean error as shown in Table 2. Mitigation with AC was 79 % for phenol and 88 % for resorcinol (contact time = 4 h, pH = 7, initial conc. = 10 ppm for both pollutants. Following the similar conditions, the removal efficiency for RHA was 76 and 85 % while for WC, it was 77 and 86 % [150]. This study established that ANN can successfully predict contaminant removal efficacy in a competitive adsorption method.

**Table 2:** Comparison of three ANN models based on AC, WC and RHA. Redrawn from Ref. [150] with the permission of Elsevier.

**Pollutant removal efficiency model**		**ME**	**MSE**	**RMSE**	**Coefficient of determination**	**Slope**	**Y-axis intercept**
Model ‘A’	Phenol	0.08	6.0	2.4	0.96	0.98	0.01
	Resorcinol	1.4	20.5	4.5	0.95	1.0	0.00
Model ‘B’	Phenol	1.5	7.5	2.7	0.93	0.89	0.06
	Resorcinol	1.3	16.2	4.0	0.97	0.98	0.01
Model ‘C’	Phenol	0.01	47.4	6.8	0.95	0.90	0.05
	Resorcinol	−1.1	8.6	2.9	0.96	0.98	0.01

The utilization of peat soil as adsorbent for mitigation of phenol in water environment was reported. ANN model was developed for elaborating adsorption process. The batch adsorption was carried out in laboratory to investigate influence of [phenol]_0_, pH, contact time and adsorbent dosage on phenol mitigation efficiency at 25 °C. Different concentrations of adsorbent dosage (50–200 g/L) were placed in 250 mL glass flask containing 100 mL of 5, 8, 10, 15 and 20 ppm of phenol. The flasks were rotated at 140 rpm for 7 h. After taking flasks out at regular interval of 1 h, they were filtered. The remaining phenol amount was estimated by spectrophotometer after color development using 0.3 mL 4-amino antipyrine and 0.3 mL potassium ferricyanide. A 3 layered FFNN trained using LM was employed. The purelin function was employed between output and hidden layer while, the tansig function was used between hidden and input layers. The input parameters were [phenol]_0_ (5–20 ppm), pH (2–10), contact time (0.5–7 h) and adsorbent dosage (50–200 g/L) while, the output variable was % mitigation of phenol. Out of 200 data sets, 100 were used for training, 50 were validated and 50 tested. The optimum number of hidden nodes was selected as 20 owing to lowest MSE of 0.00105996. This ANN model was developed using MATLAB Neural Network Toolbox version 7. The maximum removal efficiency was found at solution pH of 6 and adsorbent dosage of 200 g/L. The ideal ANN topology was 4:20:1 with R^2^ of 0.993. The close proximity of correlation coefficient to 1 is indication of efficient performance of ANN model [151]. In another study, oil palm empty fruit bunch AC was utilized for adsorptive removal of aqueous phenol. ANN was employed for elaborating optimum adsorption capacity for phenol mitigation. The input parameters included CO_2_ gas flow rate, activation times and activation temperatures while, the output parameter was optimal adsorption capacity. 40 % of data sets were tested, 40 % were trained and 20 % were validated. The ANN model exhibited extremely high accuracy with MSE = 0.0008 and R^2^ = 1 [152]. Aghav et al. modeled adsorptive eradication of phenol from water using wood charcoal. MATLAB Neural Network Toolbox version 6.5 was employed for developing the FFNN model. The input parameters included amount of wood charcoal, contact time, [phenol]_0_ and pH. The ideal ANN topology was 4:12:1 with R^2^ of 0.974 and RMSE of 1.98 [153].

The fixed bed adsorptive removal of phenol using AC was reported. The adsorption process was modeled using FFNN trained with LM backpropagation. The six input parameters included density of AC, bed height, mass of AC, particle diameter, specific surface and time while, the reduced concentration was output parameter. The activation functions utilized at output and hidden layers were purelin and tansig, respectively. The number of hidden layer neurons was varied from 1 to 20. It was found that optimum number of hidden node was 13 with correlation coefficients of 0.99 and 0.9922 for validation phase and total data set, respectively. This ANN model was developed using MATLAB Neural Network Toolbox version 8.2. The RMSE values were equal to 0.05 for validation phase and 0.044 for whole database. The maximum adsorption occurred when the values of input parameters were following: time (960.5 min), specific surface (740 m^2^/g), particle diameter (1.2 mm), mass of adsorbent (10.05 g), bed height (0.18 m) and density (1837 kg/m^3^). These results elaborated that ANN could be a robust model for explaining adsorption of phenol onto AC [154].

The efficiency of powdered AC modified with -NH_2_ groups in adsorptive mitigation of 4-CP from water was reported. They modeled adsorption process using RSM optimized by central composite design and ANN optimized by genetic algorithm. ANN model was designed using Neural Network Toolbox (MATLAB, 2013). The input variables for both models included [4-CP]_0_, adsorbent dosage, contact time and pH while, the mitigation efficiency was output parameter. The RSM model revealed highest removal efficiency of 95.35 % at adsorbent dose of 0.55 g/L, [4-CP]_0_ of 110 ppm, time of 35 min and pH of 3. The RSM model confirmed fitness between experimental data and predicted values with R^2^ of 0.9738. ANN model had tansig function at hidden layer and purelin at output layer. The ideal ANN topology was 4:10:1. ANN model predictions were extremely close to experimental data with R^2^ of 0.9851 and 0.9904 for testing and validation, respectively. It revealed removal efficiency of 95.64 % at adsorbent dose of 0.56 g/L, [4-CP]_0_ of 110 ppm, time of 35.5 min and pH of 3. The maximum adsorptive uptake of AC modified with -NH_2_ for 4-CP was 316.1 mg/g. This study revealed that stirring rate can also affect process of adsorption due to which further investigations shall be carried out for optimizing this variable to increase adsorption capability [155]. The fabrication of a composite material (ARSB-TiO_2_) was reported using titanium dioxide (TiO2) and activated biochar prepared from rice straw (ARSB) for eradicating BPA. Four ANN models trained with LMBP (Trainnlm), Resilient back-propagation (Trainrp), scaled conjugate gradient (Trainscg) and gradient descent with momentum (Traindx) were utilized for modeling of the BPA removal. MATLAB was used to design these models. The input parameters included time (minutes), ARSB-TiO_2_ dosage (gm/L) and solution pH while, the output parameter was % BPA mitigation. The correlation coefficients of ANN models increased in following order: Traindx (0.813) < Trainscg (0.904) < Trainrp (0.925) < Trainnlm (0.947). It was inferred that LMBP was most efficient algorithm for optimization of data for this ANN model [156].

An adsorbent copper impregnated activated banana tree (CIABT) was prepared using waste part of banana tree as precursor and CuCl_2_ as impregnating agent for removal of phenol. 1 g of adsorbent was used to treat 100 mL phenol solution. ANN optimized with PSO was utilized for predicting removal of phenol. 85 % of data sets were utilized for training, 10 % were validated and 5 % tested. This model was developed using MATLAB. The input parameters included adsorbent dose, pH of solution, particle size of adsorbent, contact time and [phenol]_0_. The optimum number of hidden nodes was 10. The experimental data fit closely with predicted model revealed by R^2^ of 0.99 and MSE of 0.71. Maximum mitigation of 95.99 ± 0.04 % was obtained for phenol (450 ppm), at pH 8.0, CITAB dosage of 10 g/L, particle size of 90 μ and contact time of 150 min. Hybrid ANN-PSO model achieved 86.43–99.99 % mitigation of phenol from synthetic wastewater [157]. In another study, biochar from spent tea waste was activated using sulfuric acid (STAC-H) and ortho-phosphoric acid (STAC-O) for the elimination of phenols from water. This study involved preparation of economical and safe adsorbent using waste biomass. This biomass was activated chemically for improving sorptive uptake. An FFNN model with POSLIN function at input-hidden layer and PURELIN function at hidden-output layer was employed for computing-based mathematical. This ANN model was developed using MATLAB Neural Network Toolbox version 7. The input parameters included [phenol]_0_, dosage of adsorbent, time and pH while, percentage removal of phenol was output parameter. The R^2^ between experimental and predicted results were 0.96649 for STAC-H and 0.99364 for STAC-O. It was concluded that 100 ppm of phenol could be eradicated by STAC-H and STAC-O with 3 g/L dosage, 6.9 pH within a time of 2 h; highest mitigation of 91.024 % and 93.59 % was obtained, respectively [158]. The potential of two adsorbents, including granular activated carbon (GAC) and powdered activated carbon (PAC) derived from wood for mitigation of BPA was demonstrated. A three layered FFNN with purelin at output layer and tansig at hidden layer was employed for modeling. 70 % of data sets were trained, 15 % were validated and 15 % were tested. The ideal ANN topology was 5:12:1. LM backpropagation was employed for training. MATLAB R2015a was used to design this ANN model. The R^2^ for GAC adsorbent were 0.9889, 0.9604, 0.9615 and 0.9992 for full-data, testing, validation and training. The R^2^ for PAC adsorbent were 0.9871, 0.9937, 0.9989 and 0.9839 for full-data, testing, validation and training. The optimization of ANN was done using GA, and the optimized values revealed highest removal efficiency of 89.95 % for GAC and 99 % for PAC [159]. An economical AC adsorbent using paper and pulp mill sludge (PPMS) for mitigation of phenol, 4-nitrophenol and 2-chlorophenol was prepared. The breakthrough curves for adsorption of phenolic contaminants using fixed bed column were characterized using ANN. This model was also developed using MATLAB R2015a. The input parameters included temperature, inlet concentration, bed height and liquid flow rate. Out of 146 data sets, 106 were used in training, 20 were validated and 20 tested. Two hidden layers were employed in ANN model and they both had tansig activation function. Purelin was used as activation function of output layer. The ideal topologies for phenol, nitrophenol and chlorophenol were 4:15:2:1, 4:14:12:1 and 4:5:8:1, respectively. The best validation performance for phenol, 2-CP and 4-Nitophenol was 0.0014002, 0.0029888 and 0.00074751 of MSE at 42, 47 and 40 epoch, respectively [160].

### 2.2 Photocatalytic degradation

**Fig. 5:**
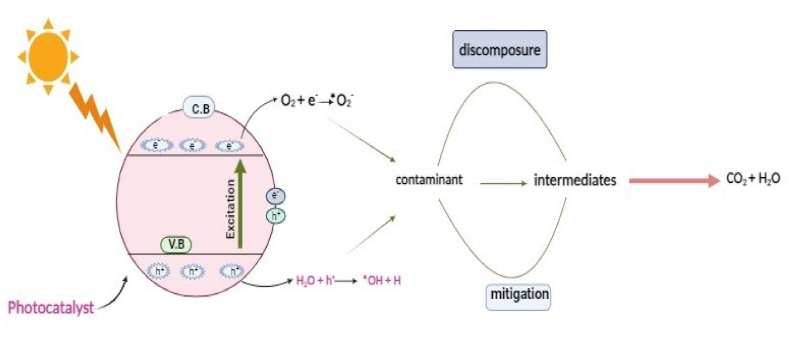
Mechanism of photocatalytic degradation of contaminants.

Photocatalysis is a technique known for transforming the organic pollutants of wastewater into water and carbon-dioxide and deleterious inorganic materials into harmless substances. A semiconducting nanomaterial capable of undergoing excitation after absorption of light is a basic necessity of photocatalysis [1]. ANNs can be trained on an experimental dataset for determining the correlations between a photocatalyst surface area, composition, shape and its activity. Subsequently, this data can be utilized to forecast performance of novel materials prior to synthesis and laboratory testing. A variety of photocatalyst-related input parameters, including material composition, morphology, surface area, doping components, etc., and reaction conditions, including light source, wavelength, temperature, pH, reactant concentration, etc., can be fed into ANNs. ANNs can forecast photocatalytic activity, which includes the production of hydrogen, the breakdown of contaminants, the efficiency of light energy transformation to chemical energy and the creation of desired products over unwanted byproducts. The general mechanism of photocatalytic treatment of contaminants has been shown in Fig. 5.

Zulfiqar et al. investigated photo-degradation of phenol with employment of TiO_2_ nanoparticles as photocatalyst. The appropriate modeling and optimization of TiO_2_ nanoparticles was done using FFNN. This ANN model was developed using MATLAB 2018a. The input parameters were pH, time (min), initial concentrations of contaminant and photocatalyst, while the output parameter was degradation of phenol. Out of 29 experimental data sets, 70 % were trained, 15 % were validated and 15 % were trained. The best ANN architecture was found to be 4:8:1. The model was trained using LMBP with 1–10 neurons in the hidden layer but M8 having eight hidden neurons gave maximum R^2^ and smallest MSE value. The maximum phenol degradation occurred at pH = 5.42, 15.21 ppm contaminant concentration, 1.75 g/L dosage of TiO_2_ nanoparticles and irradiation period of 540 min. The high pressure halogen lamp (500 W) provided photocatalytic light with intensity AM1.5 G illumination. The experimental data was found to fit perfectly with the ANN model that gave maximum value of R^2^ = 0.9988 and least RMSE = 0.8258. The benefit of ANN in this study was its capability to evaluate multiple responses to a single process. It did not need the previous understanding of the process. ANN needs less information for predicting the features of a process which makes it a robust and powerful tool for industrial applications [161]. TiO_2_ heterogeneous photocatalysis was optimized prior to its utilization in eradication of total phenolic compounds from real wastewater (tertiary and secondary wastewater) with the help of an FFNN model. STATISTICA 10.0 statistical package was used to design ANN model. The Xenon lamp (2.2 kW) provided photocatalytic light. It was equipped with filters that restricted transmittance of wavelengths lesser than 290 nm. The input parameters were photocatalyst dose, prior amount of total phenols and irradiation period while, the output parameter was photocatalytic oxidation rate of total phenols. The optimum values of input parameters were found to be 300 mg/L photocatalyst dosage, 3 mg/L total phenol concentration and 600 Wm^-2^ of irradiation time which allowed complete degradation of phenol in 180 min. The number of hidden layer neurons was varied from 1 to 14 during training of MLPs. Out of 17 experimental data sets, 14 were trained using LMBP and 13 tested. For secondary wastewater treatment, the ANN configuration 3:12:1 allowed least errors and it was most suitable for anticipating the optimum conditions for photocatalytic oxidation rate of phenolic pollutants. The R^2^ in this case was 0.972 while, the Mean Absolute Error (MAE) was 1.877. For tertiary wastewater treatment, the ideal ANN architecture was 3:4:1 with R^2^ = 0.983 and MAE = 1.232 [162].

AC-ZnO nanocatalysts were utilized for Fenton-Like photo-degradation of phenol. The photo-degradation of phenol was carried out at pH = 5 (HCL buffer) with 0.05–0.8 g AC-ZnO photocatalyst and 200 ppm of phenol solution in presence of hydrogen peroxide (2–8 mM). The light source was solar radiation measured using Apogee pyranometers (MP-100, USA). The optimal conditions for phenol degradation were 90 min of solar irradiation, pH = 5,0.2 g/L of nanocatalyst and 6 mM hydrogen peroxide. The experimental results were modulated using ANN model with MSE = 0.00526 and R^2^ = 0.998, respectively. Out of 98 experimental data sets, 62 were used in training, 18 were validated and 18 were tested. The ANN model comprised of 5 input parameters including pH, hydrogen peroxide concentration, solar irradiation period, prior amount of phenol and nanocatalyst dose while, the output parameters were uptake ability and degradation efficiency. This ANN model was trained with LM backpropagation algorithm and was developed using MATLAB Neural Network Toolbox version 8.5.1. The number of hidden neurons were varied from 3 to 18 and it was revealed by least MSE value of 0.0721 that nine neurons should be chosen as appropriate number of hidden layer neurons. So, the most accurate ANN topology had 5:9:2 configuration [163]. In another study, the degradation of phenol was demonstrated using Cu/TiO_2_ photocatalyst and an ANN model was utilized for predicting the efficiency of photo-degradation. The input parameters were amount of phenol, time period and photocatalyst dosage while, the phenol mitigation efficiency was an output parameter. The ideal ANN architecture was 3:5:1 that efficiently predicted the photocatalytic degradation of phenol with R^2^ = 0.978 [27]. The ANN modeling of p-cresol photodegradation was reported by Abdollahi et al. The photodegradation was performed by ZnO with 6 W UV-A lamp. The input parameters were pH, irradiation time, amount of photocatalyst and concentration of cresol while, the degradation percentage was treated as output parameter. Out of 38 experimental data sets, 25 were trained, 6 were tested and 7 validated. ANN model was trained with learning algorithmic programs that included Batch Propagation (BP), Quick Propagation (QP), Incremental Backpropagation (BBP) and LM. ANN models were developed using Neural Power software version 2.5. The ideal ANN topology for LM, BP, BBP and QP were 4:11:1, 4:8:1, 4:18:1 and 4:10:1, respectively. The comparison of these models revealed that QP gave the best performance with RMSE = 2.31 and R^2^ = 0.98 for validation data [164].

**Fig. 6:**
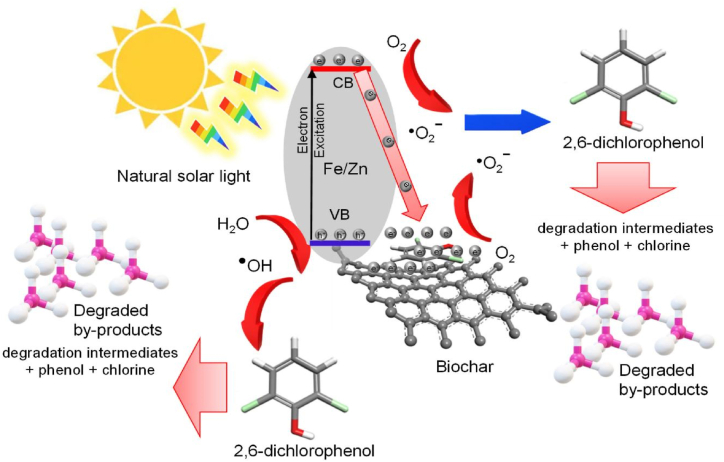
Fe/Zn@biochar adsorbing 2-6-DCP followed by photocatalytic degradation. Reprinted from [23] published under Creative Commons CC-BY 4.0.

The photodegradation of 2-nitrophenol was investigated in presence and absence of Ag/S/TiO_2_ with the help of an FFNN-GA model trained using back-propagation gradient descendant algorithm. They also examined interaction of K_2_S_2_O_8_ (PDS) and hydrogen peroxide in solution containing photocatalyst. The input parameters were amount of hydrogen peroxide and PDS while, 2-nitrophenol degradation efficiency was output parameter. Out of 17 experimental data sets, 13 were employed in training, 2 were used for validation and 2 were tested. The ideal ANN architecture was 2:4:1. The R^2^ values of 0.9877 and 0.9954 for the ANN models in the absence and presence of photocatalysts, respectively are in appropriate agreements with experimental data. This ANN model was optimized with GA for finding optimum values of PDS and H_2_O_2_ that were revealed to be 172.1 ppm and 80.9 ppm, respectively. The photodegradation decreased when the amount of PDS or H_2_O_2_ was increased from optimum values. The average degradation was 97.1 % (at optimum conditions) after 45 min of solar irradiation [165]. A novel photocatalytic material, Fe/Zn@biochar, was prepared for removal of 2,6-dichlorophenol (2,6-DCP) from petrochemical wastewater. A three layered FFNN trained with Levenberg-Marquardt was applied to predict 2,6-DCP mitigation efficiency as shown in Fig. 6. The ANN computations were performed using MATLAB (R2015a) software. The input parameters included pH, dosage of photocatalyst (mg/L), [2,6-DCP]_0_ (mg/L) and irradiation time (hour) while, the output parameter was 2-6-DCP eradication (%). The ideal ANN topology was 4:10:1. The ANN prediction accuracy was revealed by MSE = 5.56e-22 and R^2^ = 0.967. Over 90 % of 2,6-DCP could be removed under pH = 2.74, [2,6-DCP] = 130 mg/L and Fe/Zn@biochar dose = 168 mg/L in about 4 h, according to the ANN-based optimized condition. The price projected from the unoptimized photocatalytic system was sixteen percent higher than the optimum condition's total cost of $7.70/m^3^. The reduction in costs can be attributed to the optimization of the irradiation period, the lowering of the reactor's volume, and the sizes of related equipment, such as the mixer and pump. The analytical, chemical, electrical, and part replacement expenses can all be reduced by the ANN-optimized condition [23].

**Fig. 7:**
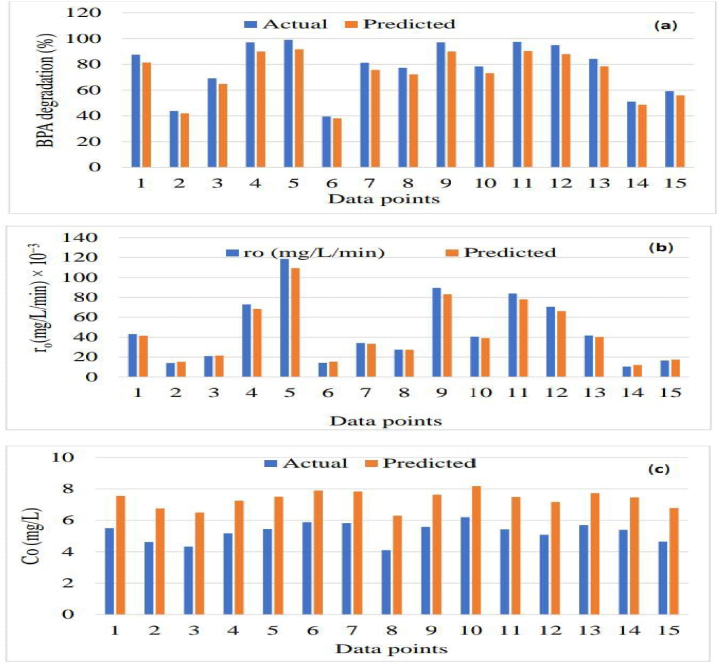
(a) Comparison between MPLNN predicted BPA removal and actual values (b) Comparison between MPLNN predicted BPA removal rate and actual values (c) Comparison between MPLNN predicted initial BPA amount and actual values. Reprinted from [167] published under Creative Commons CC-BY 4.0.

Photovoltaic system was coupled with reverse osmosis (RO) for increasing chlorophenol (CP) rejection from wastewater with minimum energy requirements. ANN model designed using MATLAB was utilized for predicting retenate and permeate concentrations of RO for CP mitigation. The input variables were water recovery rate, temperature, feed flow rate, feed concentration and pressure while, the retenate and permeate concentrations were two output parameters. Seventy-five percent of data sets were employed for training of ANN while, twenty-five percent of data points were used for validation. The ideal ANN architecture was 5:3:2. It was revealed that LM was best choice as it predicted permeate and retenate concentrations with R^2^ = 0.971 and R^2^ = 0.999, respectively. 91 % of CP degradation occurred at temperature of 40 °C, initial CP concentration of 7 × 10^−3^ kmol/m^3^, water recovery rate of forty percent, 9.713 atm of prior pressure and feed flow rate of 10^−4^ m^3^/s [166]. In another study, the dependence of photocatalytic mitigation of BPA on the textural characteristics of photocatalytic material was reported. The photocatalyst TiO_2_ was modified using iron and erbium. They prepared 200 mL solution that contained 10 ppm of BPA and 200 mg of Er and Fe modified titanium dioxide. The photocatalytic mitigation was performed under UV light source and finally, the resulting solution was purified with the help of membrane filters of cellulose acetate and HPLC was used for its analysis. MPLNN, EQGPR and CSVMR were utilized as configurations for modeling of this experiment for demonstrating relationship among potential of modified TiO_2_ in mitigation of BPA and its textural features. The input parameters included size of crystallite, pore diameter, pore volume and specific surface area. The output variables were initial BPA concentration, BPA removal and rate of BPA removal. The ideal MPLNN topology was found to be 4:13:3 because the actual values of BPA removal, rate of BPA removal and initial BPA amount were consistent with MPLNN predicted values as shown in Fig. 7. In case of MPLNN, the R^2^ and RMSE values for training were found to be 0.902 and 4.2 while, for testing the R^2^ and RMSE were 0.992 and 4.6. The EQGPR model could not give satisfactory prediction of output variables as it had R^2^ and RMSE values of 0.763 and 15.819. At each data point, the actual and predicted values slightly matched revealing inefficiency of EQGPR for modeling of photocatalytic removal of BPA. The CSVMR model could not be proven robust as it had R^2^ and RMSE values of 0.397 and 19.591 [167].

The factors affecting the octylphenol (OP) removal from surface water using solar irradiation reported. The importance of each factor regarding concentration of p-OP was demonstrated. The advanced MPLNN and conventional multiple linear regression analysis were utilized for mathematical modeling of effect of artificial and real UV-light on ultra-pure water solutions of synthetic OP. The input parameters were pH, solar UV, [Fe^3+^], solar natural, initial concentration of OP, [HCO_3_^−^], temperature of exposure, [NO_3_^−^], MP-UV 56033091, [H_2_O_2_], time of exposure and LP-UV 56001721. The output variable was concentration of OP remained after photocatalytic degradation of synthetic OP. Out of 137 data sets, 20 percent were used for testing and 80 % were trained. The ideal ANN topology was found to be 12:4:1 based on lowest ratio of S_test_/S_training_. The results of MPLNN (89 % coefficient of determination) were appreciably greater than result of regression analysis (50 % coefficient of determination). It was found that when pH is less acidic, the OP degrades rapidly because it inappropriately contributed to extremely large weight (0.1723) at hidden neuron 2 and appropriately contributed to little weight (0.0008) at hidden neuron 3. Oxygenated H_2_O also contributed to breakdown of OP because it inappropriately contributed to little weight (0.0260) at hidden neuron 2 and appropriately contributed to larger weight (1.7898) at hidden neuron 3. The contribution of carbonate ion was less in contrast to nitro. The carbonate ion appropriately contributed to negative weight (−0.3295) at hidden neuron 2 and inappropriately contributed to smaller negative weight (0.0741) at hidden neuron 3 while, the nitro ion appropriately contributed to negative weight (−0.0755) at hidden neuron 2 and appropriately contributed to positive weight (0.1519) at hidden neuron 3. The availability of Fe^3+^ ions inhibited the photocatalytic decomposition of OP because it inappropriately contributed to larger weight (0.3437) at hidden neuron 2 and appropriately contributed to larger weight (0.0255) at hidden neuron 3 [168].

The photocatalytic mitigation of 4-CP using titanium nanotubes (TNT) was investigated with consideration of validation, optimization, selection, pre-processing and assembly of two layered FFNN where the input parameters were time, pH, initial concentration of 4-CP and photocatalyst dosage while, degradation of 4-CP was output variable. This ANN model was developed using MATLAB version 7.10.0 (R2010a). The ideal ANN topology was 4:8:1. The ANN modeling revealed remarkable agreement among predicted and actual values as it exhibited correlation coefficient of 0.985. The main focus of this work was to find optimum values of input parameters that would increase the response. The ANN predicted optimum values to be 42 ppm of 4-CP, 212.51 ppm of TNT and 3.38 of initial pH for photocatalytic reaction carried out for 240 min which were in close proximity to experimental values. Among four parameters, initial 4-CP concentration and time were most significant having relative importance of 35 % and 31 % [169].

Oladipo et al. studied photocatalytic removal of phenol with employment of Fe_3_O_4_/coffee residue (MCC) as photocatalyst. The MCC was prepared by pouring 50 mL of sodium hydroxide in 100 mL of Fe_2_SO_4_.7H_2_O and FeCl_3_ at 70 °C in three neck flask for 1 h under N_2_ flow. The fabricated dark ppt. of Fe_3_O_4_ was washed, separated and dried. It was ultrasonicated in distilled water for 30 min and then glutaraldehyde and dried CC were added to suspension at 25 °C. After 1 day, MCC was collected, washed, dried and calcined for ultimately obtaining Fe_3_O_4_/coffee residue. Photocatalysis was performed using compound parabolic concentrator (CPC). The solution flow rate was 1.5 mL/min in a continuous closed cycle. 100 ppm of phenol was fed into solar reactor and reaction time was adjusted to UV intensity of 30 W/m^2^. A three layer FFNN was used for mathematical modeling of phenol mitigation. Out of 28 data sets, 60 % were used for training, 20 % were validated and 20 % tested. The ideal ANN topology was 3:2:2. The high correlation co-efficient of 0.991 was quite close to 1 which revealed that ANN is suitable to elucidate complex photocatalysis. MCC fully removed phenol within initial 100 min of solar radiation at pH 5. It also significantly removed catechol and hydroquinone (aromatic intermediates) [170]. The phenol oxidation in water with TiO_2_ NPs merged with photoelectro-Fenton process utilizing Mn^2+^ as catalyst was reported. The effect of UV irradiation on oxidizing potential was studied using 6W UV-B and UV-A lamps. The ANN modeling was used due to complexity of combined photocatalytic and photoelectro-Fenton process. The kinetic aspects of complex process including mechanisms of PEF/TiO_2_, mass transfer and absorbed radiation were difficult to be solved by equations but ANN modeling proved to be appropriate. The input variables were initial Mn^2+^ and phenol concentration, applied current, initial pH and oxidizing time. The output parameter was removal efficiency of phenol. This ANN model was coupled with GA for finding optimal operating conditions and developed using MATLAB version 7.6. Out of 106 data sets, 20 were used for testing, 56 were trained and 20 validated. The ideal ANN topology was 5:12:1. The ANN model revealed that R^2^ for validation, testing and training was 0.978, 0.985 and 0.990, respectively. The MSE for validation, testing and training was 5.902, 8.365 and 2.868. All input parameters strongly affected the phenol oxidation but effect of Mn^2+^ concentration was more pronounced. The influence of each independent variable was found to be in order of: [Mn^2+^] >pH > [phenol] >time > applied current. The PEF/TiO2 process eliminated phenol within 3 h, yielding oxidation efficiency of 78 % [171].

**Fig. 8:**
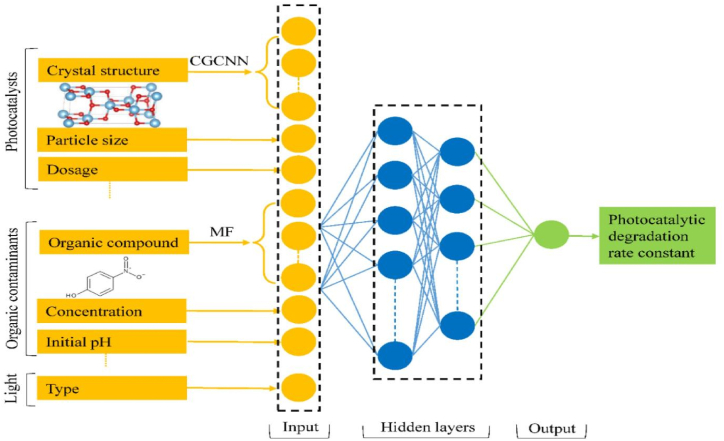
ANN model for photocatalytic degradation of phenolic pollutants. Reprinted from [175] published under Creative Commons CC-BY 4.0.

Haiqi et al. investigated the photocatalytic mitigation of phenol from crude oil wastewater. They employed ZnO/Fe_2_O_3_ prepared using sol-gel process in a batch reactor under solar irradiation. 120 mL cylindrical batch reactor was used for photocatalysis. MPLNN was used for predicting efficiency of process. The input parameters were dosage of photocatalyst, pH, initial concentration of phenol and irradiation time. The output variable was % phenol degradation. Out of 26 datasets, 15 % were used for testing, 70 % were trained and 15 validated. The ideal ANN topology of 4:5:1 predicted phenol eradication with great accuracy as it exhibited coefficient of determination (R) of 0.999. This demonstrated strong correlation between actual and predicted values. The relative order of importance of input parameters were: irradiation time > [phenol] > photocatalyst dose > pH [172]. The removal of phenol from synthetic water using nano-powder of TiO_2_ immobilized on light expanded clay aggregates was reported. FFNN based models were employed for predicting kinetic constant of phenol treatment and efficiency of photocatalytic reactor. Input parameters consisted of UV lamp intensity, pH, [TiO2], [initial phenol] and retention time. Out of 225 data sets, 205 were used for training and 120 were tested. Vesta software package was used to implement ANN model. The ideal ANN topolgy was 5:6:4:2 which demonstrated that optimal ANN model was found when correlations for testing and training were 0.97 and 0.99 and RMS errors were 0.051 and 0.023, respectively [173]. Hosts of micro-porous aluminosilicate including HEU type, FAU type and HZSM-5 (MFI type) were utilized for encapsulating zinc oxide clusters. ANN models were developed using MATLAB (version 7.8.0) for predicting the efficiency of synthesized catalysts towards catalytic degradation of 4-nitrophenol. HZSM-5 is more efficient adsorbent than clinoptilolite and zeolite. The input parameters were pH, prior amount of 4-nitrophenol and irradiation period while, the percentage degradation was output parameter. Out of 72 experimental data sets, 49 were utilized for training purposes, 13 were validated and 10 were employed in testing. The ideal ANN topology for modeling ZnO-ZSM-5 capability in mitigation was 3:6:1 with R^2^ = 0.982. This model predicted the order of importance of input parameters in photodegradation as: pH (52.39 %) > irradiation period (35.87 %) > prior amount of contaminant (11.74 %) [174]. The general ANN modeling of photocatalytic degradation of nitrophenol is provided in Fig. 8.

The ultraviolet (UV)-Fenton oxidation process was employed for m-cresol degradation using BiVO_4_. An ANN model developed using MATLAB included 6 input parameters i.e, *m*-cresol concentration, initial pH, reaction time, catalyst quantity, H_2_O_2_ dosage, and Fe^2+^ dosage. The ideal topology of model was 6:22:22:1. The ANN model projected a maximum removal rate of 87.12 %, validated by an efficiency of 86.26 % achieved through experiments under the derived optimal conditions: substrate concentration of 50 mg/L initial pH of 3.66, reaction time of 29.80 min, catalyst quantity at 0.11 g/L, H_2_O_2_ dosage at 1.40 mg/L, and Fe^2+^ dosage at 16.09 mg/L. The *R*^2^ values for the testing and training sets were 0.94 and 0.96, respectively, accompanied by an RMSE of 6.37 for the testing sets and 5.25 for the training sets [176]. Delnavaz et al. utilized nano titania immobilized on concrete as photocatalyst for treatment of phenol contaminated water. They irradiated the TiO_2_ immobilized on photocatalytic reactor by UV-A lamps. The hydroxyl radicals were generated that treated phenolic synthetic wastewater. They predicted removal efficiency through ANN modeling. The input parameters included [initial phenol], [TiO_2_], distance of UV lamp from concrete, intensity of UV lamp and pH. The pH of solution and initial phenol concentration had pronounced effect on degradation efficiency. The ideal ANN topology was 5:6:4:1. The values of R^2^ obtained for training and testing were 0.99 and 0.98 and the percentages of RMSE obtained for training and testing were 29 % and 51 %, respectively. The transfer functions of this model were Hyperbolic Tangent, Sigmoid and Gaussian for hidden layer 2, output layer and hidden layer 1, respectively [177].

The wavelength of light source was included in input parameters. The effect of kind of irradiating light on removal efficiency of BPA was studied. Silver modified zinc oxide particles were employed as photocatalysts. They were initially treated with (3-Glycidyloxypropyl)trimethoxysilane (GLYMO) in order to reduce formation of aggregates and to decorate Ag/ZnO surface with organic functional groups. Then, radical polymerization of acrylic acid in availability of a cross-linker synthesized Ag/ZnO-PAA composites. The direct interaction of bare Ag/ZnO and PAA was not desirable owing to steric hindrance caused by GLYMO. When Ag/ZnO nano-agglomerates are physically trapped among entanglements, the photocatalyst remains stable. Interaction among COOH group of PAA chains and free tail of GLYMO can possibly lead to gel formation. They used two hidden layers in two ANN models for predicting removal efficiency of BPA. The input parameters were [BPA], pH and light wavelength. The ideal ANN topologies were 3:8:10:1 with R^2^ of 0.99922 and 3:10:10:1 with R^2^ of 0.99985 [178,179].

Ghanbary et al. prepared TiO_2_ NPs for removal of 4-nitrophenol. Sample solutions of 4-NP were sonicated for 5 min prior to irradiation. After particular time intervals, 5 mL sample was taken and analyzed at 400 nm using UV–Vis spectrophotometer. In the range of 0–60 ppm, a linear relationship occurred between absorbance and 4-NP concentration with R^2^ = 0.9991. They used 3 layered FFNN for modeling the photo-catalytic process. Matlab 7.8 (2009R) mathematical software with ANN toolbox was used to perform ANN calculations. The input parameters were removal time, [4-nitrophenol]_0_, intensity of UV light and [Nano TiO_2_]_0_. The eradication percentage was selected as output variable. Out of 147 data sets, 75 were used for training, 36 were tested and 36 validated. The ideal ANN topology was 4:14:1 that validation R^2^ of 0.9925 [180].

### 2.3 Comparison of ANN with other AI techniques in mitigation of phenols

The effectiveness of ANN and RF was compared to predict photocatalytic degradation of phenol in a cascade photocatalytic backlight reactor. The TiO_2_ particles having 10–15 nm size and 3.37 eV bandgap were utilized as photocatalysts. In this study, three layered perception ANN model was designed. It was trained with LM backpropagation algorithm. The input parameters were photocatalyst concentration, [phenol]_0_, pH and treatment time while, the dependent variable was remaining phenol concentration. The hidden layer transfer functions were examined with tangent sigmoid and log sigmoid transfer functions, due to which it was necessary to scale the input data. However, in random forest (RF) method, the input data is not scaled and therefore output data is not scaled either. This leads to initial scaling of data within range of −1 to 1 for creating an ANN model, which is then again reverse-processed to original state. It made the comparison of outputs of ANN and RF possible. RF input and output parameters were similar to that of ANN. The perfect RF model was chosen on basis of certain parameters including the number of decision trees (n_tree_), number of independent variables that are randomly selected (m_try_) and node size. 192 samples were used to feed RF and ANN models. For development of an ANN model, 15 % of data sets were validated, 15 % were tested and 70 % trained. The number of hidden layer neurons were varied from 1 to 21 for obtaining accurate ANN model. The best results were achieved with 15 neurons in the hidden layer, therefore the ideal ANN topology was 4:15:1. For developing RF model, the 162 experimental data sets (85%) were trained and 29 (15 %) were tested. The RF model with 200 trees, 4 m_try_ and 3 node size produced the best results. With the treatment time of 180 min, pH of 9, TiO_2_ concentration of 91.5 g/m^2^ and [phenol]_0_ of 50 ppm, the remaining amount of phenol as predicted by ANN model was determined to be 8.16 mg/L, demonstrating high accuracy with R^2^ = 0.9996 and MSE = 0.36. The RF model predicted the remaining amount of phenol to be 10.1 mg/L with R^2^ = 0.9972 and MSE = 1.36 mg/L at TiO_2_ concentration of 90.56 g/m^2^. Hence, it was deduced that ANN predicted the removal of phenol with somewhat better accuracy than RF [109].

Another study was carried out for comparative modeling of the phenolic dyes degradation by SnO_2_/Fe_3_O_4_ photocatalysts using RBFN, ANFIS and back propagation neural network (BPNN). The tin oxide NPs were deposited on iron oxide NPs by core-shell method. 0.5 g of CTAB, 0.5 g of Fe_3_O_4_, NH_4_OH and CH_3_COOH were strongly stirred in distilled water. After 1 h of stirring, particular quantity of SnCl_2_.2H_2_O was added to mixture and stirred. After 12 h, NPs were collected by magnet, washed with water and ethanol and dried in an oven. The photocatalytic light sources were three 8 W UV-A lamps at 365 nm. In this research, MATLAB 2010a was used to devise and train the networks. The input parameters were stirring intensity, UV radiation intensity, catalyst concentration and initial phenol red concentration while, the percent phenol red removal was output parameter. The maximum R^2^ values obtained for RBFNN model with 0.8011 for validation, 0.9158 for total data and 0.9976 for training data. The maximum R^2^ values were achieved for BPNN when hidden layers’ number was 3 and they had 20:30:20 neurons. The R^2^ value for training, validation and total data sets were 0.9986, 0.9718 and 0.9879, respectively. The ideal ANFIS model had squash ratio of 1.25, accept ratio of 0.5, reject ratio of 0.15 and range of influence of 0.75. This ANFIS model had R^2^ = 0.9023 that predicted the phenol red removal less efficiently than RBFN and BPNN. These results revealed that BPNN has better prediction ability than RBFN and ANFIS because there was good agreement between experimental and predicted data. The order of prediction efficiency was BPNN > RBFN > ANFIS [116].

Singh et al. utilized RBFN, Multilayer perception network (MLPN), Generalized regression neural network (GRNN), SVM and gene expression programming (GEP) models for predicting chlorophenol mitigtion from aqueous solution using coconut shell carbon. The main purposes behind this study were to investigate effects of input variables on adsorptive removal of CP and to compare the prediction abilities of these five models. The modeling algorithms were implemented in MATLAB. The four input variables were pH, temperature, contact time and amount of adsorbate while, the remaining % of CP was output variable. The ideal architecture for MPLN model was 4:4:1 that had R^2^ values of 0.951, 0.938 and 0.940 for training, validation and testing data, respectively. The ideal RBFN topology was 4:24:1 and it gave comparable values of R^2^ to that of MPLN with 0.960 for training, 0.939 for validation and 0.943 for testing. There were 2 units in the summation layer and 320 units in the pattern layer of the ideal GRNN model. Nonetheless, the GRNN model's predictive capacity was inferior to that of the other two ANN variations, as evidenced by the R^2^ values of 0.994, 0.826, and 0.800 for training, validation, and testing, respectively. 199 square vectors from the support vector machine model were used. The values of 0.001, 788.76, and 1.973 were found to be the best SVM parameters, which included the slack variable, penalty parameter, and kernel-dependent parameter. The SVM model demonstrated sufficient correlation with the experimental data, as seen by the R^2^ values of 0.990, 0.910, and 0.881 for training, validation, and testing, respectively. Less transparency in the results and a lack of link between the input and output were the SVM model's drawbacks. The training, validation, and testing R^2^ values for the GEP model were 0.896, 0.881, and 0.883, respectively, indicating that the model's performance was below that of the ANN and SVM models. The limitation of GEP model was time consumption during selection of optimal values of model parameters. The prediction ability of these five model in removal of chlorophenol was: RBFN > MPLN > SVM > GRNN > GEP. The better performance of RBFN was attributed to its ability to overcome limitations including slow convergence rate and non-linear weight update. The modular network orientation and unsupervised learning features make RBFN an effective technique for quick and robust prediction [181,182].

Another study compared the performance of ANN and SVM in predicting the adsorptive removal of phenol in Rotating Packed Beds (REP) using activated carbon. MATLAB (2015) software was used to develop AI models. Adsorption capacity was the output parameter, and the input variables were gravity factor, initial phenol concentration, liquid spray density, and contact time. A 4:13:1 design was chosen for a three-layered FFNN. The ANN model displayed an MSE of 0.0139 and an R^2^ score of 0.9986. The values of the kernel-dependent parameter, the penalty parameter, and the slack variable for the optimal SVM were 0.25, 16384, and 0.50, respectively. The SVM model displayed an MSE of 0.0268 and an R^2^ score of 0.9966. These results showed that the ANN model did a little better job of predicting the outputs [101].

A comparative study between ANN and generalized decay-function (GEDF) was made for predicting adsorptive removal of phenol and o-cresol using activated date palm biochar (DPBC). MATLAB (R2017a) was used to train AI models for adsorption process. Fixed-bed breakthrough column tests were performed to carry out adsorption with operating conditions of 5–50 ml/min flowrate, 10–40 cm bed depth and 10–100 mg/L initial concentration. The GEDF model demonstrated the R^2^ values of 0.9213 and 0.9387 for phenol and o-cresol at 30 ml/min flow rate, 100 mg/L initial concentration and 13.3 cm bed height. Similarly, at these conditions the ANN model showed R^2^ values of 0.9880 and 0.9886 for phenol and o-cresol, respectively. ANN model was better than GEDF and it ranked the input parameters in following order: initial concentration > flow rate > time > bed depth [183].

Suditu et al. compared the predicting ability of ANN and response surface methodology (RSM) in adsorptive removal of Bromocresol Green (BCG), a phenolic dye used as coloring agent and pH indicator. The input parameters were amount of adsorbent (Activated Carbon), contact time and BCG concentration while, the output parameter was % of adsorbed BCG. Using RSM, the optimal values of these input variables were adsorbent amount = 1.58 g/L, contact time = 62.74 min and BCG concentration = 5.135 E−03 g/L that provided 39.92 % efficiency. This information pointed out that RSM is not much efficient in finding better solutions. The ANN model optimized using Bacterial Foraging Optimization (BFO) demonstrated that ideal topology had two hidden layers, with 4:33:22:1 architecture. This ANN model revealed high accuracy between experimental and predicted values as R^2^ values for training and testing data were 0.962 and 0.961, respectively. More than 99 % efficiency was achieved using ANN model proving that traditional processes can be improved when better strategies are applied [184]. The mitigation of 2-CP using coconut fibers carbon as adsorbent was compared using ANN, linear partial least square (PLS) and non-linear PLS models. The accuracy of models was found to be in order of: ANN > non-linear PLS > linear PLS. The R^2^ for ANN, linear PLS, non-linear PLS was 0.96,0.87, 0.88 and while, relative error of prediction (REP) was 7.98, 10.19 and 9.88, respectively [185]. In another study, the adsorptive removal of phenol was modeled and optimized using RSM, ANN and ANFIS. The tetraoxophosphate V acid (H_3_PO_4_) impregnated waste corncob was utilized as adsorbent. The correlation coefficients of RSM, ANN and ANFIS were 0.9430, 0.9998 and 0.9998 respectively. It showed that ANFIS and ANN were more efficient than RSM in modeling dephenolization process. Moreover, ANFIS-GA and ANN-GA optimization resulted in maximum percentage of phenol mitigation a 92.34 % and 92.44 % under optimum conditions [186].

The removal of a phenolic pollutant, 4-chlorophenol (4-CP), modeled with ANN, from wastewater using a mixed microbial consortium in Airlift packed bed bioreactor has been studied. This reactor removed 85.3 % of 4-CP at concentration of 1 mg/L and contact time of 96 h. The ANN models with 2:3:1 and 2:4:1 had minimum MSE and maximum correlation (R^2^_physical_ > 0.999) for 4-CP mitigation. ANFIS model was utilized as a model for removal of 4-CP from wastewater using a heterogeneous Fenton like system comprising of persulfate and oxalic acid combination. Though, this method of 4-CP removal had 99.6 % efficiency but ANFIS predicted the accuracy between experimental data with R^2^ value of 0.98. SVM model for electrochemical removal of 4-CP was also designed and it had R^2^ value of 0.892. Hence, the prediction ability of these three models in eradication of 4-CP from wastewater was in order of: ANN > ANFIS > SVM [187,188].

The eradication of a phenolic compound, Pyrocatechol, from wastewater was demonstrated using nanocellulose-based platelet shaped gels. The optimization attempts for predicting optimal removal conditions were made using RSM and ANN. The input parameters were pH, temperature, [Pyrocatechol]_0_ and bioadsorbent dosage, while the output parameter was % mitigation efficiency. The maximum removal efficiency 89.6 % was achieved at optimal values of pH = 3, temperature = 25 °C, bio-adsorbent dosage of 50 mg and initial pyrocatechol concentration of 100 ppm. The ANN model demonstrated high accuracy among predicted and experimental values with R^2^ value of 0.954 and SSE of 10.27 while, the prediction of RSM with R^2^ value of 0.931 and SSE of 13.25 was somewhat less accurate than ANN. The high precision of ANN can be attributed to its capability of approximating non-linear systems. On the other hand, RSM is restricted to second-order polynomial. ANN does not need standard experimental designs for computing a model [189]. Khomeyrani et al. compared the predicting ability of ANFIS, RSM and GRNN for para-nitrophenol (PNP) removal using CaAl-layered double hydrooxide-loaded magnetic graphite carbon nitride (CaAl-LDH/g-CN@Fe_3_O_4_) nanocomposites). The input variables were [PNP]_0_, sonication time, nanocomposite dose and temperature while, the output variables were mitigation efficiency and adsorption capacity. The maximum eradication efficacy of 88.8 % and adsorption capacity 21.13 mg/L would be achieved if optimum values of input variables are adsorbent dose of 10.24 mg, sonication time of 11.8 min and temperature of 38.45 °C. They found that ANFIS model provided high accuracy among predicted and experimental values than GRNN and RSM, with R^2^ value of 0.999 and RMSE of 0.0082. They also compared these three models for predicting removal efficiency of PNP using Mg/Co/Al/LDH-GO-MWCNT nanoadsorbent. The input parameters were adsorbent mass, PNP concentration, pH, temperature and sonication time while, the output variable was % removal of PNP. The maximum eradication of 96 % was achieved at optimal values of input parameters that were adsorbent dose of 8.40 mg, PNP initial concentration of 17.02 mg/L, temperature of 55.32 °C, pH of 5.55 and sonication time of 15.10 min. The prediction ability of three models was found in order: ANFIS > GRNN > RSM [190,191].

On reviewing the literature, it is inferred that mostly. ANNs have better predicting ability than ANFIS, RF, SVM, RSM, GEDF and GEP for predicting the removal of various phenolic contaminants such as phenol, ortho-cresol, chlorophenols and phenolic dyes. However, the predicting ability of ANFIS for 4-nitrophenol removal is more than ANN.

### 2.4 Challenges and future prospective

ANN has attracted a lot of interest because of its numerous applications in several sectors and has shown itself to be a potent tool for solving real-world issues. One of the key problems in the adsorption process for eliminating phenolic pollutants from water is precisely predicting and controlling the adsorption behavior of the contaminants on the adsorbent material. Understanding the complicated interactions between pollutants and adsorbents, as well as the impact of numerous environmental conditions on the process, is required. Furthermore, the adsorbent material's regeneration and reusability is a key problem in the adsorption process. ANN can assist in resolving these issues by offering a tool for modeling and improving the adsorption process, as well as forecasting the performance of various adsorbent materials under variable circumstances. The significance of ANN for the photocatalytic degradation of phenolic contaminants in water lies in their ability to model and optimize the complex interactions between the contaminants, photocatalysts, and light sources. However, some challenges during this process include accurately predicting the degradation efficiency under varying environmental conditions, optimizing the photocatalyst composition and properties for enhanced performance, and understanding the kinetics of the degradation reaction. The scalability of the process and its practical application in large-scale water treatment systems are also significant challenges. A neural network model can represent discrete interactions between multiple inputs and multiple outputs, but it is a black box and structural model. In other words, the ANN model only provides the connection between the input and output, but it cannot provide information about the problems that occur during the removal of phenol from wastewater. Tuning of the ANN model is an important problem, which affects the learning process and directly impacts the performance of the model. In some cases, the expected process predicted by a neural network may differ from the actual result. For example, rapid changes in operating conditions and water quality can cause ANNs to make incorrect predictions. One challenge in using ANN technology to remove phenol from wastewater is the selective elimination of phenol without affecting other compounds present in the water. The ANN algorithm must be able to differentiate between phenol and other chemicals to ensure effective and targeted removal. From the literature, it is noticed that there is less data available on ANN for cost approximation, conservation, and crisis management future research that should work on this aspect [192].

## 3 Conclusions

As toxic compounds that endanger the safety of our environment, phenolic pollutants from water have been the subject of extensive research aimed at mitigating their presence. These contaminants degrade the quality of the water, endangering both humans and aquatic life. Various methods have been employed to remove poisonous, hazardous, and deleterious phenols from aquatic systems, including adsorption and photocatalysis. These approaches must be modeled and optimized to be highly successful and reasonably priced. ANNs, a method based on artificial intelligence (AI), have been examined for their ability to predict the elimination of phenolic pollutants from water. Due to their ability of identifying patterns between input and output in a complex non-linear process, ANNs have been the most widely used AI tool for modeling these processes. Even though ANN is a black box, efforts are being made to elaborate the process that is being examined. The relative influence of each independent input variable on the intended output may also be ascertained using ANNs. The basis of the ANN model is the correct selection of parameters such as the number of neurons per layer, learning rate, algorithm, and transfer function. ANN models can predict removal of different kinds of phenol with correlation coefficient >0.99. It has been noticed that mostly ANN has better predicting ability than ANFIS, RF, SVM, RSM, GEDF and GEP for predicting the removal of various phenolic contaminants such as phenol, ortho-cresol, chlorophenols and phenolic dyes. However, the predicting ability of ANFIS for 4-nitrophenol removal is more than ANN. In this review paper, the literature from the past 14 years has been reported to reveal the potential of ANN to model the photocatalytic and adsorptive degradation processes in order to predict the removal of phenols in the given reaction conditions. In general, findings of this work exhibit the crucial role of the ANN model in the optimization of process parameters for remarkable eradication of pollutants. From this review, it could be concluded that the feed forward network has been most widely employed for the prediction of phenol degradation among different categories of ANNs. For training, Levenberg-Marquardt backpropagation algorithm is widely used in the existing literature. Numerous ANN models have been implemented on MATLAB software. In water purification systems, meta-heuristic optimization algorithms such as GA and PSO are sometimes used to optimize the outputs of ANN method. Regrettably, a few number of studies have been published using optimization algorithms that combine ANN approaches with other techniques for mitigating phenolic pollutants. Furthermore, additional study at both the lab and field sizes is required to comprehend the intricate treatment of phenol-contaminated water with a range of effect parameters. Investigations into the mechanisms behind the main issues with phenol removal are also necessary. Water treatment solutions that are more effective may come from hybrid ANN models, which incorporate the best features of two or more AI models. In order to improve the prediction of phenol elimination, researchers had to attempt integrating ANN with other AI models.

## Data availability statement

No data was used for the research described in the article.

## CRediT authorship contribution statement

**Shahzar Hafeez:** Writing – original draft, Validation, Investigation, Formal analysis, Data curation. **Ayesha Ishaq:** Writing – original draft, Validation, Investigation, Data curation. **Azeem Intisar:** Writing – review & editing, Supervision, Project administration, Methodology, Investigation, Conceptualization. **Tariq Mahmood:** Writing – review & editing, Software, Formal analysis, Conceptualization. **Muhammad Imran Din:** Writing – review & editing, Visualization, Resources. **Ejaz Ahmed:** Writing – review & editing, Visualization, Project administration. **Muhammad Rizwan Tariq:** Writing – review & editing, Visualization, Supervision. **Muhammad Amin Abid:** Writing – review & editing, Visualization, Supervision, Resources, Project administration.

## Declaration of competing interest

The authors declare that they have no known competing financial interests or personal relationships that could have appeared to influence the work reported in this paper.

## List of abbreviations:

CP: Chlorophenol
ANN: Artificial Neural Network
RO: Reverse Osmosis
EPA: Environmental Protection Agency
SVM: Square vector machine
ANFIS: Adaptive-neuro fuzzy inference system
GA: Genetic Algorithm
BPA: Bisphenol A
AC: Activated Carbon
STAC-O: Ortho phosphoric acid activated biochar spent tea waste
STAC-H: Ortho sulfuric acid activated biochar spent tea waste
R^2^: coefficient of determination/correlation coefficient
MSE: Mean square error
RMSE: Root mean square error
FFNN: Feed Forward Neural Network
RSM: Response surface methodology
LMBP: Levenberg-Marquardt Backpropagation
NPs: Nanoparticles
